# SPARTIN: a Bayesian method for the quantification and characterization of cell type interactions in spatial pathology data

**DOI:** 10.3389/fgene.2023.1175603

**Published:** 2023-05-18

**Authors:** Nathaniel Osher, Jian Kang, Santhoshi Krishnan, Arvind Rao, Veerabhadran Baladandayuthapani

**Affiliations:** ^1^ Department of Biostatistics, University of Michigan, Ann Arbor, MI, United States; ^2^ Department of Computational Medicine and Bioinformatics, University of Michigan, Ann Arbor, MI, United States; ^3^ Department of Electrical and Computer Engineering, Rice University, Houston, TX, United States; ^4^ Department of Radiation Oncology, University of Michigan, Ann Arbor, MI, United States; ^5^ Department of Biomedical Engineering, University of Michigan, Ann Arbor, MI, United States

**Keywords:** Bayesian modeling, digital pathology, hierarchical strauss models, spatial point processes, tumor-immune microenvironments

## Abstract

**Introduction:** The acquisition of high-resolution digital pathology imaging data has sparked the development of methods to extract context-specific features from such complex data. In the context of cancer, this has led to increased exploration of the tumor microenvironment with respect to the presence and spatial composition of immune cells. Spatial statistical modeling of the immune microenvironment may yield insights into the role played by the immune system in the natural development of cancer as well as downstream therapeutic interventions.

**Methods:** In this paper, we present SPatial Analysis of paRtitioned Tumor-Immune imagiNg (SPARTIN), a Bayesian method for the spatial quantification of immune cell infiltration from pathology images. SPARTIN uses Bayesian point processes to characterize a novel measure of local tumor-immune cell interaction, Cell Type Interaction Probability (CTIP). CTIP allows rigorous incorporation of uncertainty and is highly interpretable, both within and across biopsies, and can be used to assess associations with genomic and clinical features.

**Results:** Through simulations, we show SPARTIN can accurately distinguish various patterns of cellular interactions as compared to existing methods. Using SPARTIN, we characterized the local spatial immune cell infiltration within and across 335 melanoma biopsies and evaluated their association with genomic, phenotypic, and clinical outcomes. We found that CTIP was significantly (negatively) associated with deconvolved immune cell prevalence scores including CD8+ T-Cells and Natural Killer cells. Furthermore, average CTIP scores differed significantly across previously established transcriptomic classes and significantly associated with survival outcomes.

**Discussion:** SPARTIN provides a general framework for investigating spatial cellular interactions in high-resolution digital histopathology imaging data and its associations with patient level characteristics. The results of our analysis have potential implications relevant to both treatment and prognosis in the context of Skin Cutaneous Melanoma. The R-package for SPARTIN is available at https://github.com/bayesrx/SPARTIN along with a visualization tool for the images and results at: https://nateosher.github.io/SPARTIN.

## 1 Introduction

While the staining and examination of tissue samples has been a ubiquitous medical practice for decades, it has only been relatively recently that high-resolution pathology images of such stainings have begun to be explored through the lens of formal quantitative models (Heindl et al., 2015). A considerable amount of work has thus far been put into building and training statistical and machine learning models capable of accurate classifications of overall tissue samples as well as subsections of tissue samples (Amgad et al., 2019; Lu et al., 2020; Bian et al., 2021; Negahbani et al., 2021). In the context of cancer this work, often referred to as *Histopathological Image Analysis* or *Digital Pathology*, encompasses a broad collection of methods and goals ranging from developing models to assist in the quantitative scoring and staging of cancer biopsies to the classification of cells in tumor biopsies (Komura and Ishikawa, 2018). The value of these methods have important implications for basic science and translational research. Given the precision with which such algorithms can assess biopsies at a cellular level, it is now possible to uncover structures and associations which might not immediately apparent to human pathologists. This degree of precision also allows for rigorous modeling and examination of intratumoral heterogeneity with respect to their genomic determinants and clinical characteristics (Saltz et al., 2018; Li et al., 2019a).

Several research works in the past decade provide evidence regarding the importance of this line of investigation, especially for quantifying tumor-immune cellular interactions in cancer. Pagès et al. (2010) proposed immune reaction as the seventh hallmark of cancer, and laid out different associations between various types of immune cells and outcomes of interest. Among the different types of immune cells, the most well-studied are lymphocytes. Lymphocyte infiltration is both a meaningful prognostic indicator that can help inform treatment and predict survival across different types of cancer, including in colorectal cancer (Idos et al., 2020), breast cancer (Denkert et al., 2015; Dieci et al., 2018), and melanoma (Fu et al., 2019). This has led to increased interest in computational Tumor Infiltrating Lymphocyte (TIL) assessment, a sub-field of digital pathology devoted to developing computational methods to assess lymphocyte infiltration in biopsy images. Thus far, results that have emerged from this research have provided additional evidence regarding the importance of tumor infiltrating lymphocytes and their spatial features in the assessment of pathology images. Saltz et al. (2018) specifically examined the presence of lymphocytes across biopsies in several types of cancer, and found that certain summarization metrics of lymphocyte clusters were significantly associated with survival in certain types of cancer. Lu et al. (2020) also found that various spatial features of tumor infiltrating lymphocytes present in a sample of breast cancer biopsies were significantly associated with survival both marginally and after adjusting for other factors. Further, they found that these associations actually differed by tumor subtype. In addition to this, they found that certain spatial features of tumor infiltrating lymphocytes were also significantly associated with gene expression data.

From an analytical perspective, digital pathology research can be broadly classified along two axes: the type of quantitative models, and the scale of the assessment. Quantitative models range from primarily machine-learning in nature to primarily statistical. The most obvious way this difference manifests is in the models and methods used. Machine learning approaches tend to use tools like deep learning and convolutional neural networks and focus on predictive accuracy (Khosravi et al., 2018), while statistical approaches tend to use specialized model-based spatial methods and focus more on the quantification of uncertainty (Seal et al., 2022). The scale of assessment, on the other hand, relates to how much of the biopsy is used. Some methods operate on sub-regions of the biopsy images while others operate at the level of the entire biopsy (Ertosun and Rubin, 2015; Li et al., 2019b).

In theory, these two qualities-type of research and scale of assessment-are independent. In practice, methods that use machine learning approaches tend to operate on whole biopsy images or data, while statistical approaches tend to operate on sub-regions of images, or substantially smaller biopsies as with multiplex imaging data analysis (Seal and Ghosh, 2022). There has been limited work to build off of the cell classification abilities of modern machine learning methods at the level of full biopsy imaging. There are two probable reasons for this. First, many statistical methods designed for usage with spatial data rely on the definition of a window in which the analysis occurs. For biopsies, such a window is not readily defined, except in deliberately chosen sub-regions. While such issues can be avoided by using methods that do not rely on an explicit window, this tends to introduce additional computational challenges that may not scale to the biopsy level, especially as biopsy size and resolution increases. Second, even if one were to define a singular window for the entire biopsy, applying spatial methods across the entire space may prove problematic. In addition to being computationally intensive, it is not clear that measures of various local spatial characteristics summarized at the biopsy level are able to capture the heterogeneity across the biopsy, and thus may not be informative enough to be useful in practice. For these reasons, there has been limited work in development of spatial statistical models that use cell classification data from full biopsy images. To the author’s knowledge, the most notable attempts to do so have been Li et al. (2019b)’s work which utilized data classified by the ConvPath pipeline (Wang et al., 2019).

**FIGURE 1:**
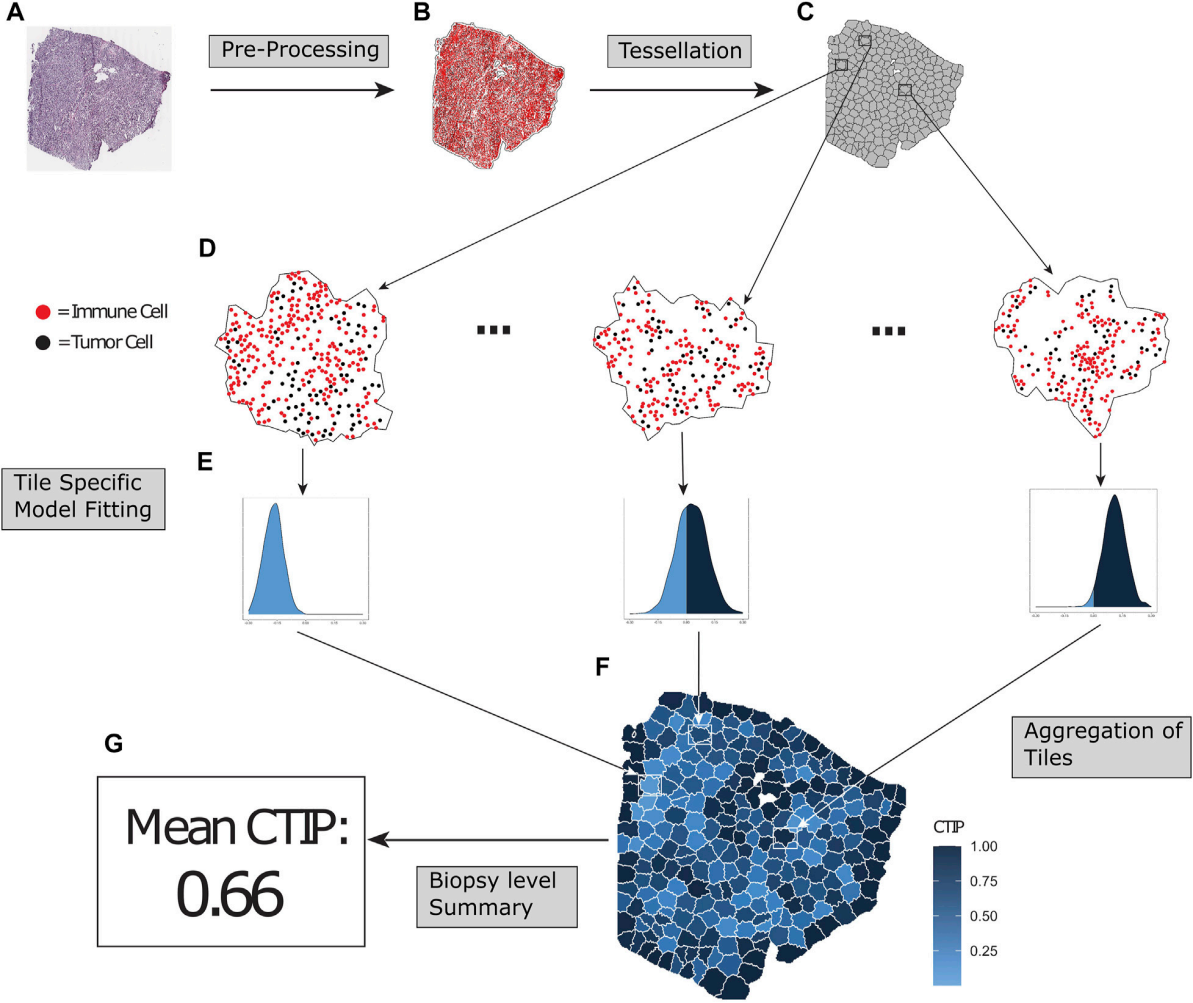
Overview of SPARTIN pipeline for single biopy image. Raw biopsy images **(A)** are preprocessed into cell types (tumor cells or immune cells, colored black and red respectively) and locations. The points are intensity thresholded, removing excess white space from the image and producing a more closely fitted window **(B)**. Next, the biopsy is tessellated into “tiles” consisting of non-overlapping sub-regions containing comparable numbers of tumor cells **(C)**. Bayesian Spatial point process models are fit on each tile **(D)**, and tile-specific Cell Type Interaction Probability (CTIP) is calculated from the resulting posterior distributions **(E)**, allowing us to capture heterogeneity in infiltration across the biopsy in a manner that can be aggregated **(F)** and appropriately summarized **(G)**.

In order to address these issues, we propose SPARTIN (SPatial Analysis of paRtitioned Tumor-Immune imagiNg) pipeline. SPARTIN is unique in the spatial pathological imaging analysis literature in that it uses spatial statistical models to assess the association between tumor cells and immune cells across an entire partitioned biopsy, rather than select sub-regions. This allows for rigorous quantification of uncertainty in the style of Li et al., 2019b while still allowing for the assessment of full images in the style of Saltz et al. (2018). We accomplish this by partitioning the cell-level imaging data of each biopsy into non-overlapping sub-regions, which can then be modeled to capture the local infiltration patterns using techniques from spatial point process theory. Spatial point processes have long been used in the domain of ecology to rigorously investigate spatial relationships between various organisms (Högmander and Aila, 1999; King et al., 2012). More recently, methods from this field have been successfully applied within the biomedical domain to functional neuroimaging and magnetic resonance imaging data (Kang et al., 2011; Ray et al., 2015). Briefly SPARTIN uses whole slide images of biopsies that are partitioned into sub-regions using an iterative clustering algorithm and a Bayesian spatial marked point process model is then fit on each sub-region (see Figure 1). We discuss the constructions and justifications behind the choice of models underlying SPARTIN in Section 2. Using our model parameters, we construct a measure of local immune cell infiltration, termed *Cell Type Interaction Probability* (CTIP) that allows rigorous incorporation of uncertainty and is highly interpretable (on a probability scale) both within and across biopsies, and can be used to assess associations with genomic and clinical features. We evaluate the performance of SPARTIN on simulated data across multiple scenarios of cell interactions (Section 3.1) and show that our models are able to reliably distinguish positive interaction from negative and null interaction under a variety of scenarios (minimum AUC 0.85, maximum 0.97).

We demonstrate the utility of SPARTIN on a Skin Cutaneous Melanoma dataset consisting of 335 biopsies obtained from The Cancer Genome Atlas (TCGA) Genomic Data Commons Data Portal (Section 3.2). Our scientific goals are to quantify and characterize local spatial immune cell infiltration within and across melanoma biopsies and evaluate their association with genomic, phenotypic, and clinical outcomes. Using local spatial infiltration patterns quantified using CTIP, we found that CTIP was significantly negatively associated deconvolved immune cell prevalence scores including *CD*8+ T-Cells and Natural Killer cells. Furthermore, average CTIP scores differed significantly across previously established transcriptomic classes in melanoma (Akbani et al., 2015). CTIP was also more significantly associated with increased hazard of death, as compared to existing measures of spatial interaction. Beyond the inverse association with survival we found that CTIP was significantly inversely associated with clinical assessment of TIL abundance. Finally, we discuss the implications of this pipeline as well as potential future directions for the work (Section 4).

## 2 Materials and methods

### 2.1 Overview of SPARTIN

The overall schematic of the SPARTIN pipeline (for a single biopsy image) is shown in Figure 1. Briefly, for each whole-slide image of a biopsy (Panel A), the smallest window that fit all cells (Tumor and Immune) in the entire biopsy is constructed by thresholding the image intensity (Panel B). This window is then divided into “tiles” via a tessellation process that fully partitions the image into contiguous sub-regions containing similar numbers of tumor cells (Panel C; zoomed in version in Panel D). A Bayesian spatial point process model is then fit on each of these tiles separately, yielding a posterior distribution of the local interaction parameter (Panel E). For each tile, this local distribution is compared to a tile-specific null distribution to compute the local CTIP value, which is then combined to summarize the overall level of interaction at the biopsy level (Panel G). Each of these steps in detailed in the ensuing sections.

### 2.2 Intensity thresholding and partition

#### 2.2.1 Motivation for partition

Each biopsy was partitioned into non-overlapping sub-regions, each of which was modeled separately as a point process. The primary motivation for structuring the analysis in this manner came from prior biological knowledge about tumor composition. Tumors are heterogeneous entities in many respects, and immune infiltration is no exception. Tumor biopsies can also be quite large relative to the scale on which interaction is defined. While we used a radius of interaction of 30 microns, some biopsies in our sample comprised tens if not hundreds of thousands of square microns of tissue. Additionally, setting aside practical computational issues with fitting a single model on an entire biopsy, such an approach would also both fail to capture spatial variability in immune infiltration and fail to allow for a more granular spatial investigation of the immune infiltration in a given biopsy. A natural way to improve over this method is to algorithmically partition the biopsy into non-overlapping sub-regions and fit models on each sub-region. Aside from better capturing the spatial variability of immune infiltration, an additional benefit of this method is that it allows for the parallelization of model fitting across the resulting sub-regions within a single biopsy. Much like selecting a quadrature when approximating an integral, calibrating the fineness of the partition entails a tradeoff between the precision with which one can assess the spatial heterogeneity within the biopsy and the computational load that a finer partition entails.

#### 2.2.2 Partition pipeline

In order to partition a given biopsy into non-overlapping sub-regions, we began with the smallest bounding rectangular window that contained all cells. We then applied an intensity thresholding algorithm in order to find the smallest possible window that still contained all cells. This was accomplished by breaking the full image up into small (∼15 microns^2^) sub-windows, and computing a smoothed cell intensity on each sub-window. Sub-windows which had a sufficiently high intensity were combined to form the overall window on which the subsequent partition took place. The final window can thus be thought of as the union of many small square windows which, due to their size, are able to fit the contours of the differently shaped biopsies and exclude areas both outside and within the biopsies where no cells are present. The algorithm takes in three tuning parameters that determine the resulting partition. The same values for all three parameters were used across all biopsies, and the values themselves were selected through empirical tuning on a subset of the total dataset. For more details on the parameters themselves, see Supplementary Section S3.

Next, we applied a voronoi tessellation to the tumor cells within the intensity thresholded window, partitioning it into tumor cell specific sub-windows. We then applied a modified version of K-means to the tumor cells, such that each of the resulting clusters was constrained to be between a pre-defined range of cell counts. Finally, each tumor cell specific sub-window within a given cluster was combined into a tile, corresponding to each of the clusters from the K-means clustering; see Figure 1 for an illustration of the process. This results in a set of tiles that fully partition the intensity thresholded window. This partition uniquely defines the membership of each of the total cells (tumor or immune) into one of the resulting tiles. Because each resulting tile has a well-defined boundary and each cell in the biopsy belongs to exactly one tile, a hierarchical Strauss model can be fit on each tile to compute a tile specific value of each parameter in the model. Most notably, this allows for the estimation of a tile-specific CTIP value, which captures the local degree of tumor cell-immune cell interaction. By modeling this parameter locally to each tile, we are able to capture not only the overall level of infiltration in the biopsy, but also the potentially spatially heterogeneous nature of the infiltration.

It is worth emphasizing at this point the modular nature of our pipeline. The clustering method does not depend on the details of the model used, and the model in turn does not depend on details of the clustering method. Either can be exchanged for a different algorithm or model without disrupting the rest of the pipeline, so long as the output of the clustering algorithm is a spatial partition of the biopsy. Further, while the value ultimately selected to summarize the local infiltration at the tile level will obviously be informed by the exact model selected, there is flexibility in the choice of this quantity as well.

### 2.3 Spatial point process models

#### 2.3.1 Data structure

Let *N* be the number of biopsies, each with *N*_*i*_ tiles. More formally, after the partition biopsy *i* can be represented by $T_{i1}\cup \cdots \cup T_{iN_{i}}$ , where each *T*_*ij*_ closed subset of $R^{2}$ . Further, let ***x***_*ij*_ = ***x***_*ij1*_ ∪ ***x***_*ij2*_, where each ***x***_*ijk*_ is a set of points in $T_{ij}\subset R^{2}$ , and *k* = 1 for tumor cells and 2 for immune cells. Then for each element of the partition of each biopsy, this data can be naturally viewed as the realization of a marked point process. Broadly speaking, marked point process models are a family of models that treat the number of points, their locations, and their discrete classification (often called their “mark”) as random. We specifically chose to model this data as a hierarchical Multitype Strauss Process. Hierarchical Strauss Processes are unique in that they allow for the modeling of both the tendency of points of different types to be generally close (positive interaction) as well as generally far apart (negative interaction).

#### 2.3.2 Model construction

The usage of a Strauss model to study spatial interaction in a marked point process is natural in this setting, since Strauss models were originally conceived for this purpose (Strauss, 1975). There are two primary model variants to consider when modeling multi-type data using the Strauss model: the hierarchical variant and the symmetric variant. These models are superficially similar, but differ in important ways. Broadly speaking the symmetric variant models the interaction between the different types of points jointly, whereas the hierarchical variant models the locations of certain types of points *conditional* upon the locations of other types. Using the hierarchical Strauss model thus requires an important choice for each pair of types as to which type of point will be “dominating,” i.e., conditioned upon, and thus unaffected by the other type. This choice, which must be made with *a priori* information about the nature of the process, has important consequences for the resulting model fit. Aside from the differing interpretations depending on the choice, different choices of dominating vs. non-dominating types will also result in different conditional likelihoods and thus parameter values (Högmander and Aila, 1999). It is also worth noting here that the term “hierarchical” is used differently in this context than it is often used in more typical statistical modeling. Rather than referring to mechanisms to account for dependence in observations or induce shrinkage, here the term refers to the hierarchical relationship between the dominating and non-dominating types: the locations of the dominating type do not depend on the locations of the non-dominating type, but the locations of the non-dominating type *do* explicitly depend on those of the dominating type.

**FIGURE 2:**
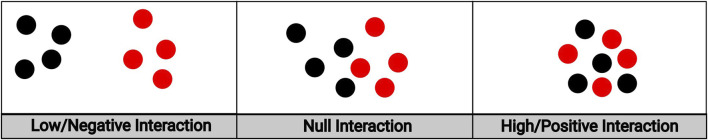
An illustration of spatial interaction between cells of different types. The leftmost panel depicts low/negative interaction; the middle panel depicts null interaction; the rightmost panel depicts high/positive interaction.

We ultimately chose to use the hierarchical variant of the multi-type Strauss model over the symmetric variant for two key reasons. First, in our imaging application treating the locations of the immune cells as conditional upon the locations of the tumor cells is *a priori* biologically plausible. It has been established that there is an interplay between a tumor and the host’s immune system whereby the immune system responds to the tumor, thus shaping its development, and the tumor in turn “escapes” the immune system and influences its response (Khong and Restifo, 2002; Zhang and Zhang, 2020). While such dynamics are no doubt of interest, this natural evolution cannot be easily inferred from a “snapshot” in time that a biopsy presents. Despite this, conditioning on the locations of the tumor cells to model the immune response is a reasonable starting point: in order for the immune system to respond, there must at the very least be a tumor to respond to. The hierarchical Strauss Model thus serves as a first order approximation to this complex interplay. Second, the hierarchical Strauss model allows for the proper modeling of positive interaction between points of different types, while the symmetric model does not. Figure 2 depicts examples of different types of spatial interaction, ranging from negative to positive. The left and center panels depict the range of interactions that can be modeled by the traditional Strauss process: either the case where cells of both types actively avoid the other (left panel) or are spatially unaffected by the other’s presence (middle panel). Because we are interested in not just these cases but the case where points of both types tend to cluster around points of the other type (right panel), the hierarchical variant is the clear choice.

The density function of the hierarchical Multitype Strauss Model with two qualitative marks (tumor and immune cells in our setting) is defined by

$$f\left(x_{1},x_{2}\right)\propto \exp\left(n_{1}\beta_{1}+n_{2}\beta_{2}+S_{R_{11}}\left(x_{1}\right)\gamma_{11}+S_{R_{22}}\left(x_{2}\right)\gamma_{22}+S_{R_{12}}\left(x_{1},x_{2}\right)\gamma_{12}\right)$$ (1)

where in addition to ***x***_1_ and ***x***_2_ as defined above, *n*_*t*_ is the number of points of type *t*, *β*_*t*_ is the first order intensity of points of type *t*, $S_{R_{tl}}\left(\cdot \right)$ counts the number of pairs of points of types *t* and *l* within *R*_*tl*_ of one another where *R*_*tl*_ is selected *a priori* based on subject specific knowledge, and *γ*_*tl*_ captures the tendency of points of type *l* to be near points of type *t*. It bears mentioning that this model can be extended to arbitrary numbers of marks (i.e., types of cells). For our current application, we have focused on tumor-immune interactions, but it would be trivial to extend the chosen model to as multiple cell types as were available for a given data set.

#### 2.3.3 Interpretation of parameters

As previously mentioned, *γ*_*tl*_ can be thought of as the degree to which points of type *l* tend to be close to or far away from points of type *t*. This allows us to distinguish between the mere relative abundance of different types of points (which is captured by the *β*_1_ and *β*_2_ parameters) and the actual spatial associations between the different types of points. This distinction is important in situations where there are substantial numbers of points of both types but no positive spatial association (and possibly a negative one). For examples, see Section 3.1 for results from the simulation study. When *t* = 1 and *l* = 2, this association is interpreted conditionally upon the locations of the type 1 points. Under the hierarchical Strauss model, *γ*_12_ ∈ (−*∞*, *∞*), with *γ*_12_ ∈ (−*∞*, 0) implying negative interaction, *γ*_12_ ∈ (0, *∞*) implying positive interaction, and *γ*_12_ = 0 implying no interaction at all. As one might intuitively expect, larger values of *γ*_12_ correspond to more positive interaction, and smaller values correspond to a more negative interaction. Due to mathematical constraints on the density, we must have $\gamma_{11},\gamma_{22}\in \left(-\infty ,0\right]$ . This constrains interaction between points of the same type to be modeled as negative or null, i.e., as in panels 1 and 2 of Figure 2. Since this constraint is unlikely to be satisfied in the context of a tumor biopsy, we set *γ*_11_ = *γ*_22_ = 0, in effect assuming no interaction between cells of the same type. Based on prior biological knowledge, we set *R*_12_ = 30 microns. The need to select values of *R*_11_ and *R*_22_ is obviated by setting *γ*_11_ = *γ*_22_ = 0.

### 2.4 Model fitting

Except in the trivial case where *γ*_12_ = 0, the normalizing constant of this distribution is computationally intractable. This motivates the usage of a pseudolikelihood formulation as outlined by Baddeley and Turner, 2000. For the density *f*(⋅) of the simplified hierarchical Strauss Model outlined in (1), define the conditional intensity function at a point *u* given *θ* = {*β*_1_, *β*_2_, *γ*_12_} and points ***x*** observed in window *A* by

$$\lambda \left(u|\theta ,x\right)=\left\{\begin{array}{cc} \frac{f\left(x\cup \left\{u\right\}\right)}{f\left(x\right)}\; & u\notin x\\ \frac{f\left(x\right)}{f\left(x-\left\{u\right\}\right)}\; & u\in x \end{array}\right.$$ (2)

Given this, the pseudolikelihood is defined as follows,

$$PL\left(\theta |x\right)=\underset{x_{i}\in x}{\prod }\lambda \left(x_{i}|\theta ,x\right)\exp\left(-\int_{A}\lambda \left(u|\theta ,x\right)du\right)$$ (3)

In essence, this provides a computationally tractable alternative to the likelihood function that can be used in inference. Specifically, the integral in the pseudolikelihood function can be easily approximated by summing over a weighted quadrature on *A*, as described in Baddeley and Turner, 2000. See Supplementary Equation S1 for additional details.

Using this approximation of the pseudolikelihood function in place of the more standard likelihood function, Bayesian analysis can proceed in the style of King et al., 2012 by simply assigning priors to the parameters of interest and using techniques to sample from non-closed form posterior likelihoods. We assigned non-informative normal priors with mean 0 and variance 10^6^ to *γ*_12_, *β*_1_, and *β*_2_. In all analysis presented below the quadrature and weights used to estimate the integral in the pseudolikelihood function was generated by the spatstat package (Baddeley and Turner, 2005). Samples from the posterior were taken using JAGS via the R2jags package (Yu-Sung et al., 2015); see Supplementary Algorithm S1 for details.

### 2.5 Cell type interaction probability

#### 2.5.1 Motivation

For ease of exposition, we suppress the subscript in *γ*_12_ parameter, and refer to it as *γ*_•_ in the following sections. *γ*_•_, *β*_1_, and *β*_2_ allow us to distinguish between the tendency of cells of different types to be spatially near one another (captured by *γ*_•_) and the relative abundance of the cells of each type (captured by *β*_1_ and *β*_2_), as well as the uncertainty around these various tendencies. However, this does not capture the difference between the observed spatial association and what one would expect to observe by chance given a particular configuration of tumor cells. Even if the locations of the immune cells are completely random and unrelated to those of the tumor cells, there is still a possibility of a configuration that may indicate positive interaction when considered in isolation. In order to properly account for this, the observed distribution of *γ*_•_ must be compared to a counterfactual distribution that captures the tendency when there is no interaction. This motivated the development of Cell Type Interaction Probability (CTIP).

#### 2.5.2 Definition

Let *π*(*γ*): = *f*(*γ*_•_| ***x***_1_, ***x***_2_) be the posterior distribution of *γ*_•_ conditional upon the observed tumor cells and immune cells, and $\pi_{0}\left(\gamma \right):=f\left(\gamma_{0}|x_{1},{\tilde{x}}_{2}\right)$ be the posterior distribution of the interaction parameter conditional upon the observed locations of the tumor cells and ${\tilde{x}}_{2}$ , a set of points distributed as a Poisson Process. Further, assume independence between the true distribution and the null distribution. Then we define the CTIP (denoted by *r*) as:

$$\;r=\iint I\left(\gamma >\gamma_{0}\right)\pi \left(\gamma \right)\pi_{0}\left(\gamma_{0}\right)d\gamma d\gamma_{0},$$ (4)

where *I*(⋅) denotes the indicator function, and both *π*(*γ*) and *π*_0_(*γ*_0_) are as defined above. By construction, the observed parameter is independent from the null parameter, since immune cells drawn from a Poisson process should not yield any information about immune cells realized under either positive or negative interaction. More concretely, by the definitional independence of ${\tilde{x}}_{2}$ from ***x***_2_, it follows trivially that $f\left(\gamma_{•},\gamma_{0}|x_{1},{\tilde{x}}_{2},x_{2}\right)=f\left(\gamma_{•}|x_{1},x_{2}\right)f\left(\gamma_{0}|x_{1},{\tilde{x}}_{2}\right)$ . This construction means that CTIP is the expectation of an indicator function, and thus can be interpreted as a probability. Specifically, it represents the probability that the observed value of *γ*_•_ is larger than what we would observe if there was truly no underlying interaction.

#### 2.5.3 Sampling from the empirical null distribution

The problem of estimating CTIP hinges on being able to sample from the distribution of *γ*_0_, since estimation of the posterior can proceed as described in Section 2.3. In order to estimate the null distribution of *γ*_0_ conditional upon the location of the observed tumor cells, we used simulation to construct an empirical null distribution. Recall that when there is no interaction, by definition *γ* = 0. This means that the hierarchical Strauss given in Eq. 1 reduces to two independent Poisson processes with intensities *β*_1_ and *β*_2_, which are trivial to simulate.

Given this, the estimation of the null distribution for a given tile proceeded as follows. First, the first order intensity of the immune cells was estimated using the standard estimator, ${\hat{\beta }}_{2}=\log\left(\frac{I_{ik}}{A_{ik}}\right)$ where *I*_*ik*_ is the number of immune cells observed in tile *k* of biopsy *i* and *A*_*ik*_ is the area in square microns of the tile. Second, *S* simulations of immune cells were generated from a poisson process with intensity ${\hat{\beta }}_{2}$ , *S* being selected *a priori*. Third and finally, each simulated set of immune cells was superimposed over the actual observed tumor cells, and samples were drawn from the resulting posterior distribution. These samples, combined across simulations 1, …, *S* approximate *π*_0_(*γ*) as previously defined.

#### 2.5.4 Computation of CTIP

Having established the ability to sample from the posterior distribution as well as the null distribution for *γ*, estimation of CTIP can be done via stochastic integration. Note that Eq. 4 is equivalent to $E_{\gamma ,\gamma_{0}}\left[I\left(\gamma >\gamma_{0}\right)\right]$ . Thus, given *P* posterior samples *γ*_1_, …, *γ*_*P*_ from the posterior *π* and *γ*_01_, …, *γ*_0*P*_ from the empirical null *π*_0_, (5) can be estimated by

$$\hat{r}=\frac{1}{P}\overset{P}{\underset{j=1}{\sum }}I\left(\gamma_{j}>\gamma_{0j}\right)$$ (5)

#### 2.5.5 Interpretation

By definition, *r* ∈ [0, 1]. As previously mentioned, *r* can be construed as the expected value of an indicator random variable with respect to the joint distribution of *π*(*γ*) and *π*_0_(*γ*_0_). This naturally yields an interpretation of *r* as a probability. Specifically, it can be thought of as measuring the posterior probability that the observed value of *γ*_•_ is larger than the value that would be observed given the observed tumor cells and immune cell intensity if there were no underlying interaction.

#### 2.5.6 Model outputs and summaries

Because models can be fit for each sub-region *T*_*ij*_ of biopsy *B*_*i*_, CTIP can also be computed for each tile. The result for each biopsy is a collection of tile specific estimations of CTIP for each biopsy, $\left\{{\hat{r}}_{i1},\ldots ,{\hat{r}}_{iN_{i}}\right\}$ for *i* = 1, *…*, *N*. These estimates are then summarized at the biopsy level into a singular value that quantifies the overall level of infiltration in the biopsy, which can be used in downstream analyses to investigate the association between biopsy level of tumor-immune interaction and other patient features of interest-see Section 3.2 for results. We chose to use the mean value, since even under the assumption of spatial association between tiles the result is an unbiased estimate of the biopsy level mean. Because the values themselves encode the local spatial association for each tile, the result is an appropriate proxy for the amount of interaction between tumor cells and immune cells at the biopsy level. We further logit-transformed the tile level CTIP values prior to computing the biopsy level mean in order to make the resulting distribution across biopsies closer to a Gaussian distribution. See Algorithm 1 for the full SPARTIN method.

Algorithm 1 SPARTIN algorithm.

**Input**: *N* biopsies
**for** biopsy *i* = 1, *…*, *N* **do**
Intensity threshold biopsy *i*
Partition intensity thresholded biopsy *i* into *N*_*i*_ tiles
**for** tile *j* = 1, *…*, *N*_*i*_ **do**
Estimate CTIP ${\hat{r}}_{ij}$ for tile *j*
**end for**
Compute ${\hat{m}}_{i}=\text{mean}\left(\text{logit}\left({\hat{r}}_{i1}\right),\ldots ,\text{logit}\left({\hat{r}}_{iN_{i}}\right)\right)$

**end for**
**return** ${\hat{m}}_{1},\ldots ,{\hat{m}}_{N}$ for usage in downstream analysis

## 3 Results

### 3.1 Simulations

#### 3.1.1 Simulation overview

To compare our model’s detection of spatial association between different cell types, we conducted simulation studies in which we tested the ability of SPARTIN to accurately identify positive interaction across a range of different simulated cell compositions and degrees of spatial association. For each of four sets of simulations, the number of simulated tumor cells and immune cells were set *a priori* at *T*_*s*_ and *L*_*s*_. These levels across the four simulations were, respectively, 20 and 50, 50 and 20, 50 and 50, and 100 and 100. Further, interaction was controlled by a parameter *ϕ* ∈ [−1, 1], with −1 indicating the most negative possible interaction using our simulation method and 1 indicating the most positive possible interaction using our simulation method. For each of the four settings, 50 data sets were simulated for *ϕ* = ±1, ±0.8, ±0.6, ±0.4, ±0.2, and 100 were simulated for *ϕ* = 0. Because the goal was classification as either positive interaction or non-positive (i.e., null or negative) interaction, we used AUC as our summary metric. This is a natural choice, since CTIP is interpreted as the posterior probability of positive interaction. As a benchmark for performance, we compared CTIP to the G-cross function, a commonly used non-parametric statistical method for assessing the spatial relationship between different types of points in marked point processes (Van Lieshout and Baddeley, 1999).

#### 3.1.2 Simulations of negative interaction

For a given combination of *T*_*s*_ and *L*_*s*_, the simulations of positive interaction and simulations of negative interaction proceeded differently. For the simulations of negative interaction (*ϕ* ∈ [−1, 0]), *T*_*s*_ tumor cells and *L*_*s*_ immune cells were simulated as independent poisson processes in regions of the window that overlapped to varying degrees. The overlap was controlled by *ϕ*, such that the processes overlapped on (100 ⋅ (1 + *ϕ*))% of the window in which the simulation occurred. Note that when *ϕ* = −1 there was no overlap, and when *ϕ* = 0 there was complete overlap and the simulation reduced to generating two independent Poisson Processes within the same window.

#### 3.1.3 Simulations of positive interaction

For the simulations of positive interaction ( $\phi \in \left(0,1\right]$ ), *T*_*s*_ tumor cells were simulated under a Poisson process. After their locations were determined, *L*_*s*_ immune cells were simulated. For each immune cell *l*_*i*_, a Bernoulli random variable *C*_*i*_∼*Bern*(*ϕ*) was drawn. If *C*_*i*_ = 1, *l*_*i*_ was simulated within 30 microns of a randomly selected tumor cell. Otherwise, *l*_*i*_ was simulated from a Poisson process. Thus, the level of interaction was again controlled by *ϕ*, with *ϕ* = 0 again corresponding to two independent Poisson Processes and *ϕ* = 1 corresponding to a situation in which all immune cells are within 30 microns of at least one tumor cell.

#### 3.1.4 Simulation assessment

Because CTIP is most naturally interpreted as a probability, in order to classify a given collection of points as indicating positive or negative interaction in practice a cutoff value must be chosen above which CTIP is considered to indicate positive interaction and below which it is not. This leads to a natural tradeoff: if the cutoff is too low, then many cases where there is no positive interaction will be incorrectly classified as having positive interaction. If the cutoff is too high, the opposite will occur. This tradeoff can be visualized across different thresholds using a receiver operating characteristic (ROC) curve, which can in turn be summarized by the area under the curve (AUC). AUC ranges between 0.5 and 1, with 0.5 indicating total inability to correctly discriminate between different outcomes and 1 indicating perfect ability to discriminate. AUC is therefore a natural choice for a metric to assess the ability of CTIP to distinguish between positive and non-positive interaction between cells of different types.

As a benchmark for performance, we examined the performance of the G-cross function in discriminating positive interaction from non-positive interaction. The G-cross function is a non-parametric estimate of the cumulative distribution functions of minimum distances from cells of one type to those of another. In this application, the G-cross function evaluated at a specific distance yields a non-parametric estimate of the probability that a tumor cell will have at least one immune cell within that distance. We chose to evaluate the G-cross function at a distance of 30 microns in order to be consistent with the modeling of CTIP.

#### 3.1.5 Results

**FIGURE 3:**
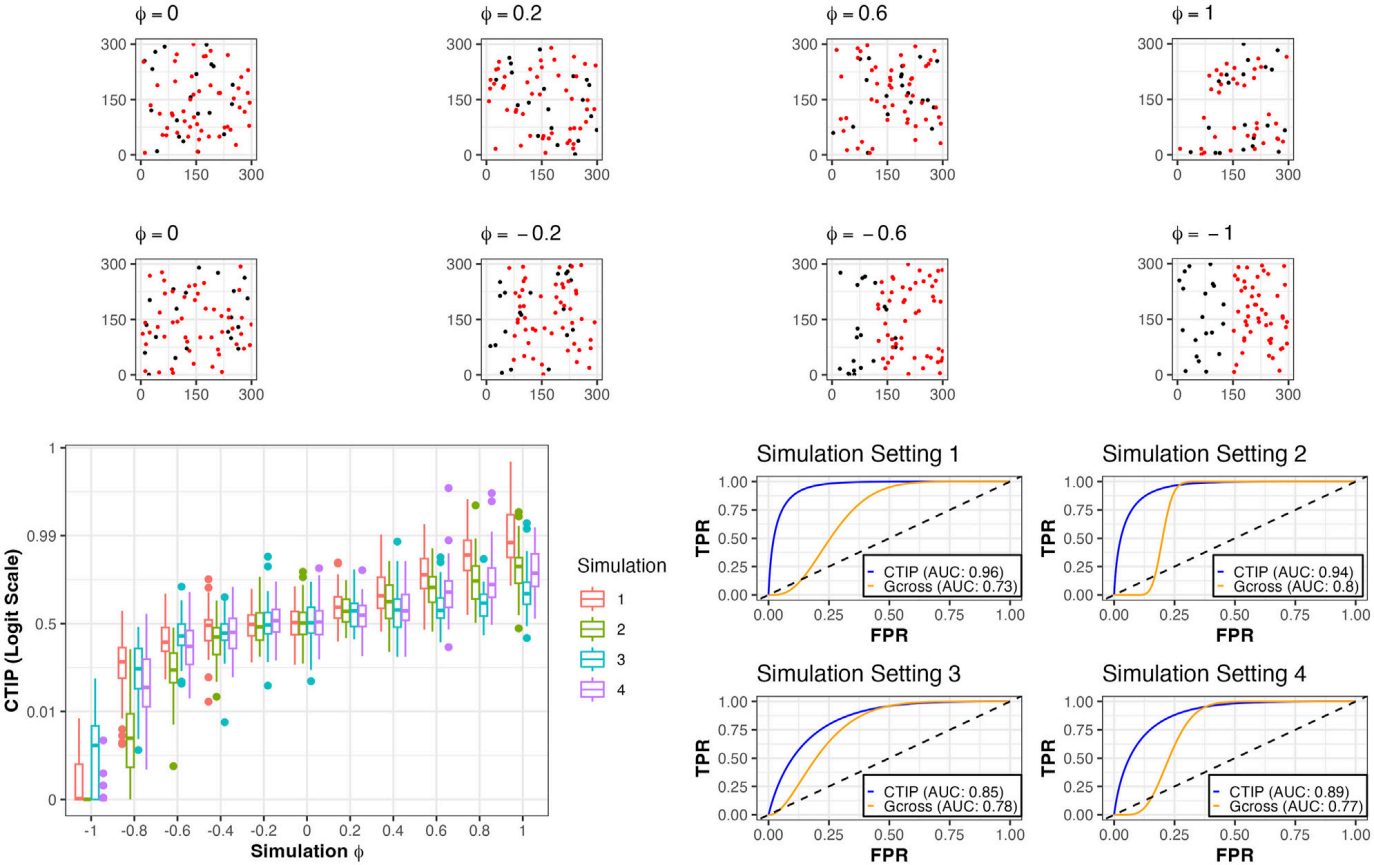
Simulated examples and results. Top row: examples of simulations of positive and negative interaction for different values of *ϕ*, all from simulation setting 1. Bottom left: box plots of CTIP across all simulated compositions for different values of *ϕ* on logit scale. Bottom right: ROC curves and associated AUC for classification (positive interaction vs. non-positive interaction) for different simulation settings and the two methods investigated (CTIP and G-cross).

For examples of simulated data as well as visualizations of results for CTIP, see Figure 3. Across the different simulation settings, accurate classification was achieved using CTIP. The top panels depict a sample of simulations across a range of interaction values, from *ϕ* = −1 to *ϕ* = 1. The bottom left panel shows the distribution of the estimated CTIP values for each set of simulations across each of the four sets of simulations and the values of *ϕ*, plotted on the logit scale. The bottom right panel shows the AUC for each of the four simulation settings across all values of *ϕ*. The results of these simulations demonstrate that CTIP reliably distinguishes between a broad ranges of interaction levels, across a similarly broad range of relative abundances in cell types. The lowest overall AUC (0.85) was achieved in simulation 3, while the highest was achieved in simulation 1 (0.96). For the G-cross function, the highest overall AUC was achieved in simulation 2 (0.8), while the lowest was achieved in simulation 1 (0.73)—see Supplementary Figure S4 in the supplementary materials for more detailed results for the G-cross function, and Supplementary Section S5 for additional simulation results examining the relative performance when model assumptions are violated.

### 3.2 Application

We applied the SPARTIN pipeline to a data set consisting of 335 high definition images of SKin Cutaneous Melanoma (SKCM) biopsies stained using hematoxylin and eosin, obtained from The Cancer Genome Atlas (TCGA) Genomic Data Commons Data Portal. SKCM is an appealing target for the investigation of immune cell infiltration for several reasons. SKCM has been shown to be particularly responsive to immunotherapy in some cases (Achkar and Tarhini, 2017; Franklin et al., 2017). It is possible that the ability to quantify immune infiltration at a large scale may allow for more detailed investigation into the scenarios in which this treatment may be most effectively deployed. It is also well established that SKCM has an unusually high mutational load amongst the various cancer types (Berger et al., 2012). The ability to quantify spatial infiltration patterns may allow for further downstream investigations into not only genomic associations of this occurrence but associations with other phenotypic and clinical outcomes as well.

#### 3.2.1 Cell classification model

##### 3.2.1.1 Whole-slide image (WSI) retrieval and obtaining training labels

WSI from 20 different patients were used as our primary training and testing data. The images were imported from The Cancer Genome Atlas (TCGA) Genomic Data Commons Data Portal, which is a repository of validated datasets from various National Cancer Institute Programs: https://www.cancer.gov/tcga. Also known as the gigapixel pathology image, one image slide contained two tissue smears stained with hematoxylin and eosin, imported in a vendor specific format. The imported image for classification is scanned at the “high” magnification level in a microscope of ×40. The pixel size for images was approximately 0.25 microns per pixel. Manual marking of a minimum of ten samples from each of the classes, i.e., Tumors, Immune, Macrophage, and other cell was performed by a pathologist on all slides. Care was taken to ensure that the samples were as diverse as possible, to account for all possible morphologies of the same cell type across all cases. In total, 1,250 annotations were made in all the images, with each annotated cell labelled by a pathologist. The reasons for the comparatively smaller labelled dataset available lie in the large size of the slide, and pathologist availability; the large size makes parsing through difficult and explains the diversity of labelled structures in each image.

##### 3.2.1.2 Whole-slide image (WSI) nucleus segmentation

Before nuclear segmentation and extraction can be performed on the WSIs, certain pre-processing steps are carried out to ensure staining uniformity across images. A representative and generalizable stain vector estimation for each of the stains being used in the image is estimated, to normalize the staining intensities across all the images. In our application, vectors from hematoxylin, eosin and residual stains were estimated from a standard image, selected by the clinician. These vectors are then applied across all images to keep the stain detection parameters uniform across the dataset (Bankhead et al., 2018).

Due to the variations in staining procedures across the dataset, it is difficult to accurately isolate whole cells on the slide, as the cytoplasmic boundaries are not well-defined due to lack of membrane staining. As heterogeneity in nuclear morphology has been shown to be a good discriminator, only the nucleus is segmented out of each cell in every image for classification purposes, using Qupath (Yuan et al., 2012). Watershed segmentation is used to obtain separated and contoured nuclei from the hematoxylin color channel of the whole slide image, in a patch-based manner (Bankhead et al., 2018). The separate hematoxylin channel is obtained by performing color deconvolution to separate out the stains used in the slide (Ruifrok and Johnston, 2001). It was observed that an average of 200,000 cells were segmented out per image, and morphological and intensity features such as spatial location, eccentricity, circularity, and stain intensities were computed. All pertinent image analysis and nuclear segmentation was performed using Qupath, an open-sourced software platform that can be used for a range of pathological image analysis applications (Bankhead et al., 2017).

A Random Forest model is a type of ensemble learning method, where the weak predictive power of multiple decision trees is aggregated to produce an accurate result (Breiman, 2001). Our four-class classifier model was developed with the pathologist labelled dataset of 1,250 morphologically diverse cells, each belonging to Tumor, Immune, Macrophage, or other cells (including cell types such as stromal cells). 5-fold cross-validation was used to assess model accuracy and the receiver operator characteristic curve AUC for each class, with proportional representation of all 4 classes ensured. After adjusting for multiple parameters, including the number of decision trees, the accuracy was obtained in the range of 87%–91%.

This step allowed for a preparation of a dataset for each cell with its spatial location using global coordinates and class of the cell, which is then used for further downstream analysis. The training, testing, and classification of cells from the WSIs was performed on MATLAB version 2017A. For visualizations of distributions of cell counts across biopsies, see Supplementary Figures S1–S3. For this analysis, only data for tumor cells and immune cells were kept to quantify the immune cell infiltration using SPARTIN.

#### 3.2.2 SPARTIN analyses of SKCM

While we are broadly grouping immune cells together for the purposes of this analysis, it is important to note that within the microevironment of melanoma there is a great deal of heterogeneity in the types of immune cells present. Tumor Infiltrating Lymphocytes (TILs) receive a disproportionate amount of attention in cancer literature due to the well-established prognostic value of the presence. However, in the context of melanoma this focus can obscure the tremendous amount of variability in the types of immune cells present in the microenvironment. Even referring to TILs as a monolith obfuscates the different cell types captured under this umbrella, many of which serve different purposes (Mukherji, 2013; Weiss, 2016; Antohe et al., 2019). The richness of the immunological aspect of the melanoma microenvironment makes it a logical target of analysis via the SPARTIN pipeline.

##### 3.2.2.1 CTIP quantification

**FIGURE 4:**
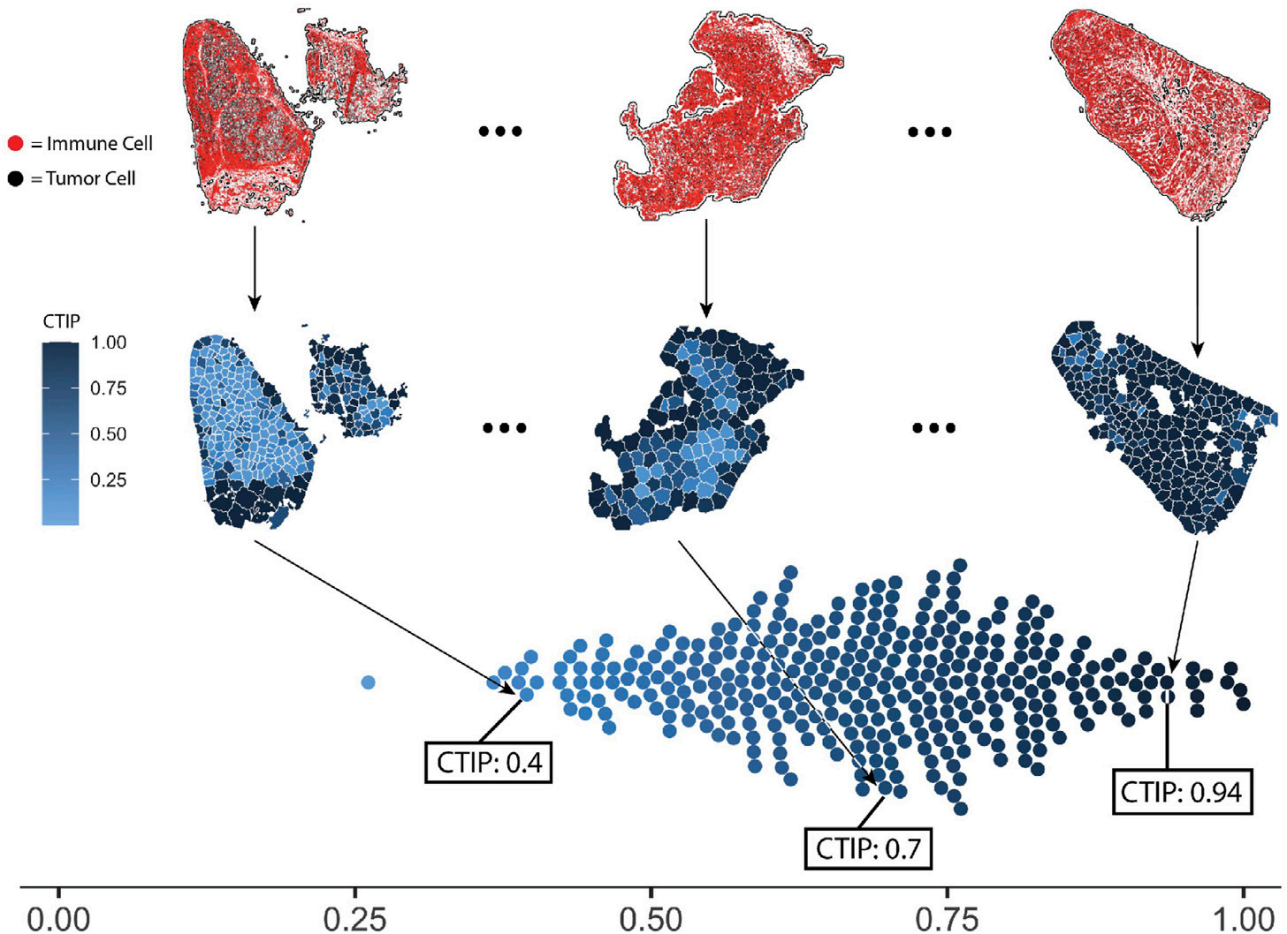
Visualization of selected biopsies, and distribution of CTIP across all biopsies analyzed. Selected examples of actual biopsies, both raw spatial data and with tessellations and CTIP by tile. The leftmost biopsy pictured has among the lowest mean estimated CTIP in the sample (0.4), while the rightmost has among the highest (0.94). The middle biopsy has average mean CTIP (0.7), but also exhibits more spatial variation than the left or right biopsy. Bottom: distribution of mean CTIP by biopsy for all biopsies investigated. This illustrates the between-biopsy variation in overall levels of infiltration.

In order to compute CTIP, models were fit in using the methods described in Section 2. The same settings were used for all biopsies across the pipeline. Each biopsy was partitioned such that the resulting tiles would have approximately 75 tumor cells per tile, with the minimum possible tumor cells per tile being 50 and the maximum being 100. For an analysis of the sensitivity to the biopsy level estimation of CTIP to the number of tumor cells per tile as well as the clustering algorithm used, see Supplementary Section S6. The CTIP quantifications are shown in Figure 4 Panel A wherein we have highlighted three biopsy images (top row). In the middle row, the color of each tile indicates the value of CTIP estimated for that tile. More varied colors across a given biopsy are indicative of more spatial variation in CTIP, and thus infiltration. CTIP was summarized at the biopsy level by taking the empirical mean across all tiles for a given biopsy, which are plotted in the bottom row. As can be seen, the range of CTIP was 0.26–0.99 with a median CTIP value of 0.69 and an interquartile range of 0.19. This points to the fact that SKCM has high level of immune cell infiltration and these results are consistent with the conventional wisdom that melanoma is a generally more immunogenic cancer relative to other variants (Mukherji, 2013; Fu et al., 2019).

#### 3.2.3 Genomic associations

**FIGURE 5:**
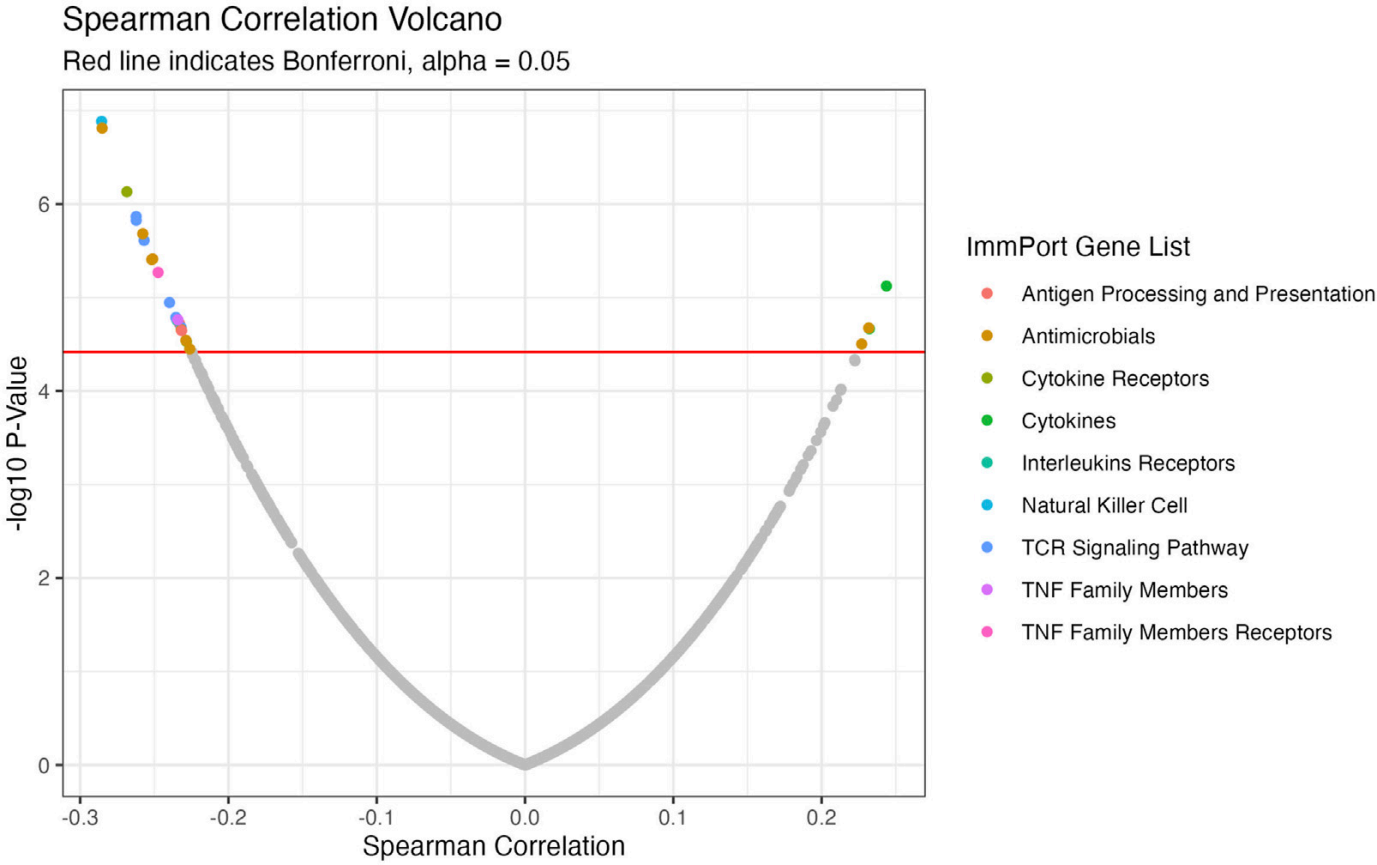
Volcano Plot of Spearman correlations between CTIP and gene expression values. Negative log_10_ *p*-values plotted against Spearman Correlation for ImmPort genes. Colors indicate ImmPort Gene List, which are the collections provided by ImmPort to organize genes into groups of similar function; Red line indicates Bonferroni correction-see Table 1 for full list of significant genes.

We conducted a focused analysis on CTIP and genes that are associated with immune activity. Bhattacharya et al., 2018 have collected and classified a list of 1,793 unique genes associated with various aspects of human immune activity. Notably, this includes genes related to processes that involve or are directly related to immune cells of interest including CD8^+^ T-cells, Natural Killer cells, and B cells. Of these, gene expression data was available for 1,305 genes across 330 patients. We assessed the univariate association between biopsy level mean logit-CTIP and these genes using Spearman correlation. The advantage of Spearman correlation as opposed to the more standard Pearson correlation is that the former does not assume a linear relationship between the underlying variables of interest. Such assumptions can be problematic, particularly when there is no strong reason *a priori* to believe the relationship between the two variables is of a particular form. However, like Pearson correlation Spearman correlation is defined to lie in [−1, 1], with each extreme indicating the same directionality and strength of association as Pearson Correlation. After applying a Bonferroni correction, we found that 28 genes were significantly associated with CTIP. See Figure 5 for a volcano plot of results; for the complete list of genes, see Table 1. Most notably, mean logit-CTIP was most significantly negatively associated with CD244, a gene associated with increased cytotoxicity in natural killer cells (Agresta et al., 2018). In addition, mean logit-CTIP was significantly negatively associated with expression of Cathepsin S, a gene associated with immunosuppression in the tumor microenvironment, and C-C motif chemokine receptor 8 (CCR8), a gene related to immunosuppressive T regulatory cells (Fuchs et al., 2020; Weaver et al., 2022).

**TABLE 1:** Full ImmPort Analysis Results. ImmPort Genes significantly associated with average logit biopsy level CTIP after Bonferroni correction, alpha = 0.05.

Gene	Group	Spearman Correlation	*p*-value
CD244	Natural Killer Cell	−0.29	≪ 0.0001
CTSS	Antimicrobials	−0.29	≪ 0.0001
CCR8	Cytokine Receptors	−0.27	≪ 0.0001
CD3G	TCR Signaling Pathway	−0.26	≪ 0.0001
PTPRC	TCR Signaling Pathway	−0.26	≪ 0.0001
TLR8	Antimicrobials	−0.26	≪ 0.0001
ITK	TCR Signaling Pathway	−0.26	≪ 0.0001
TLR3	Antimicrobials	−0.25	≪ 0.0001
STAT1	Antimicrobials	−0.25	≪ 0.0001
TNFRSF9	TNF Family Members Receptors	−0.25	≪ 0.0001
EREG	Cytokines	0.24	≪ 0.0001
LCK	TCR Signaling Pathway	−0.24	<0.0001
PIK3CG	TCR Signaling Pathway	−0.24	<0.0001
ERAP1	Antigen Processing and Presentation	−0.24	<0.0001
TNFSF14	TNF Family Members	−0.23	<0.0001
IL6R	Interleukins Receptors	−0.23	<0.0001
CIITA	Antigen Processing and Presentation	−0.23	<0.0001
ICOS	TCR Signaling Pathway	−0.23	<0.0001
DES	Antimicrobials	0.23	<0.0001
APLN	Cytokines	0.23	<0.0001
IFIH1	Antimicrobials	−0.23	<0.0001
RFX5	Antigen Processing and Presentation	−0.23	<0.0001
LYZ	Antimicrobials	−0.23	<0.0001
CXCR6	Cytokine Receptors	−0.23	<0.0001
CRABP1	Antimicrobials	0.23	<0.0001
CYBB	Antimicrobials	−0.23	<0.0001

We also investigated the association between CTIP values and tumor deconvolution data in order to assess the association between the presence of specific immune cell types and overall tumor-immune interaction. Because the specific immune cell types were not ascertained in the data collection, deconvolution algorithms can offer an estimation of the relative prevalence of different types of immune cells. We obtained data from the Tumor Immune Estimation Resource 2.0 (TIMER 2.0): http://timer.cistrome.org/. Using data from TIMER2.0, we examined the association between the prevalence of different types of immune cells including CD8^+^ T cells, Natural Killer cells, and B cells with biopsy level mean logit-CTIP. Among the available options from TIMER 2.0, we decided to use the MCP-counter algorithm (Becht et al., 2016) based on the analysis of Sturm et al., 2019, since it was judged to be most effective in detecting the presence and prevalence of the most relevant types of immune cells. We investigated the association of the score of each type of immune cell estimated by MCP-counter with biopsy level mean logit-CTIP using Spearman correlation (as done previously for gene expression). Significance was assessed using the standard test of statistical significance of Spearman correlation.

**FIGURE 6:**
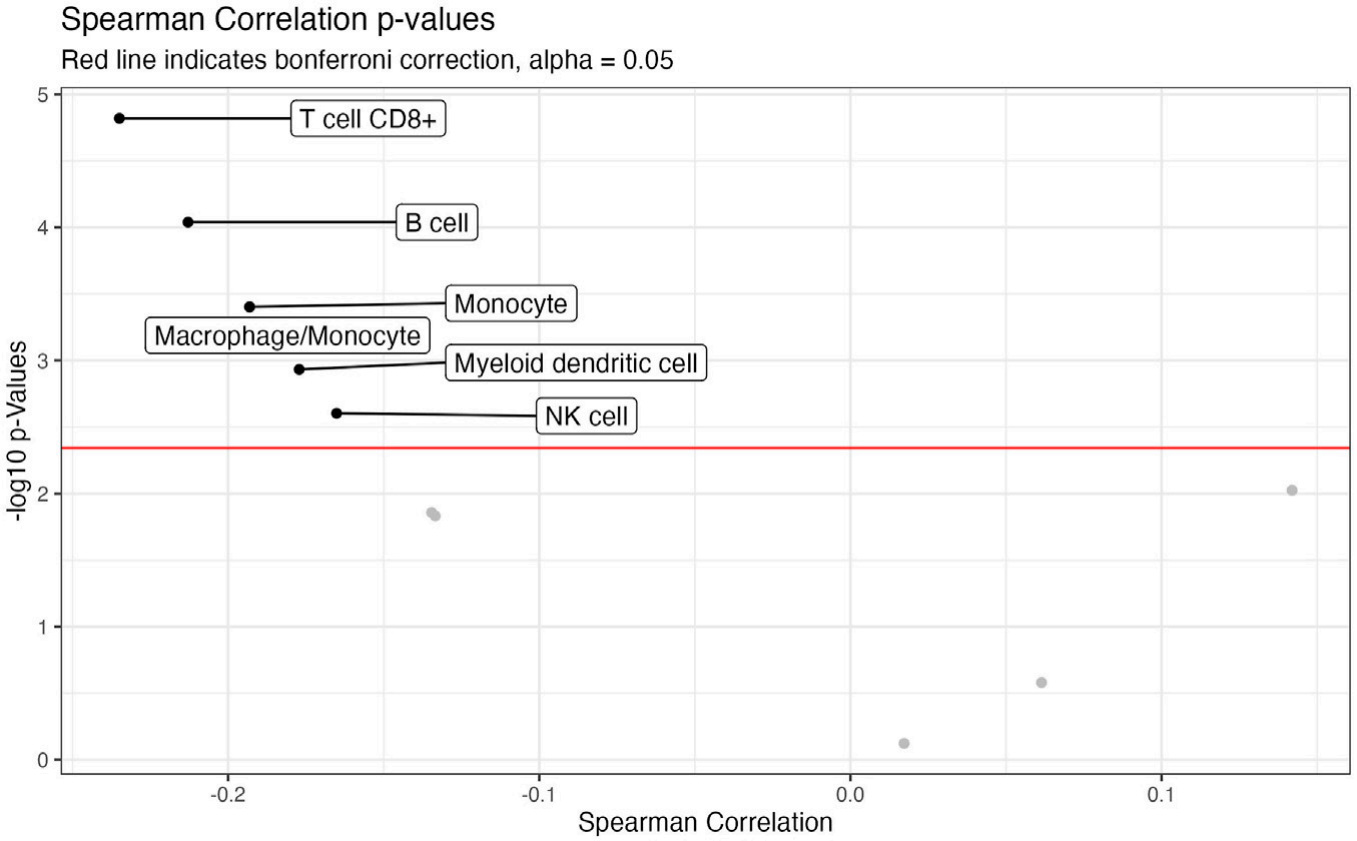
Volcano plot of association with deconvolution data. Negative log-10 *p*-values plotted against Spearman Correlation between CTIP and cell type prevalance scores estimated by MCP. Red line indicates Bonferroni correction, alpha = 0.05.

After applying a Bonferroni correction (*α* = 0.05), we found that six different immune cell scores as computed by MCP-counter were significantly negatively associated with biopsy level mean logit-CTIP: CD8^+^ T cells, B cells, Monocytes, Macrophages, Myeloid Dendritic Cells, and Natural Killer cells. No cell types were significantly positively associated with biopsy level mean logit-CTIP after the Bonferroni correction, though the magnitude of the positive association with Cancer Associated Fibroblasts (CAFs) is notable, and while not statistically significant still highly consistent with a truly positive underlying association between biopsy level CTIP and prevalence of CAFs. See Figure 6 and Table 2 for full details on specific cell type score associations. CD8^+^ T cells, B cells, and Natural Killer cells are generally associated with better prognosis in SKCM. This would imply, perhaps somewhat counterintuitively, that higher mean logit-CTIP would be expected to be associated with poorer survival, and lower levels of pathology assessment of lymphocyte infiltration. However, this is precisely what we observed, as discussed in next Section.

**TABLE 2:** Associations with cell prevalence. Cell type associated with average logit biopsy level CTIP. *p*-values are presented uncorrected; see Figure 6.

Cell Type	Spearman Correlation	*p*-Value
T cell	−0.13	0.014
T cell CD8^+^	−0.23	<0.0001
cytotoxicity score	−0.13	0.015
NK cell	−0.17	0.0025
B cell	−0.21	<0.0001
Monocyte	−0.19	0.0004
Macrophage/Monocyte	−0.19	0.0004
Myeloid dendritic cell	−0.18	0.0012
Neutrophil	0.02	0.75
Endothelial cell	0.06	0.26
Cancer associated fibroblast	0.14	0.0094

#### 3.2.4 Association with phenotypic and clinical outcomes

##### 3.2.4.1 Transcriptomic classes and pathology asessments

**FIGURE 7:**
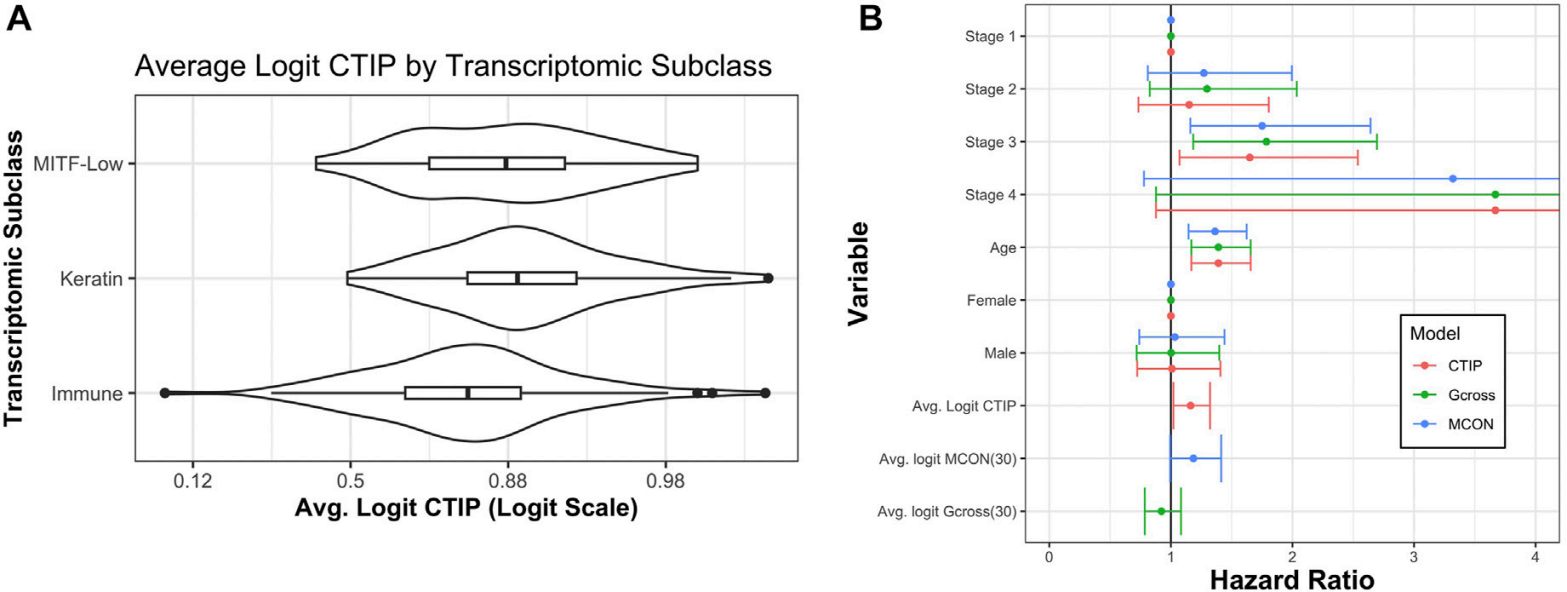
Average CTIP by transcriptomic subclass and overall survival hazard ratios. **(A)** Average logit-CTIP by transcriptomic class according to Akbani et al. (2015). **(B)** Hazard ratios from Cox Proportional Hazards models. Points at 1 indicate reference category. Age was standardized by subtracting the mean value and dividing by the standard deviation. Logit-CTIP was significantly associated with increased hazard of death (*p* = 0.019), while normalized logit-MCON was non-significantly associated with an increased hazard of death (*p* = 0.057) and normalized logit-G-cross was non-significantly associated with a decreased hazard of death (*p* = 0.32).

Akbani et al. (2015) also identified three transcriptomic classes by applying consensus hierarchical clustering techniques to gene expression data from 1,500 genes: the “immune” subclass, the “keratin” subclass, and the “MITF-low” subclass. Most notably for our current application, the immune subclass was characterized by overexpression of genes associated with T cells, B cells, and Natural Killer cells. Of the patients classified using these methods, 235 were present in our data set. Of these 235, 114 (49%) were in the immune subclass, 78 (33%) were in the keratin subclass, and 43 (18%) were in the MITF-low subclass. Figure 7 demonstrates a clear visual difference between the immune subclass and both the MITF-low and keratin subclasses. In order to determine if these apparent differences were statistically significant, we analyzed the differences in average logit-CTIP between each pair of classes using a standard two-sided *t*-test, and found that the mean logit-CTIP in the immune class was significantly lower than either the keratin or the MITF-low subclass (*p* < 10^–5^ and *p* = 0.011, respectively). We found no significant difference between the mean logit-CTIP in the MITF-low subclass and the keratin subclass (*p* = 0.21). This is highly consistent with the results of the deconvolution analysis, which suggest that the degree of abundance of such cells are inversely associated with mean logit-CTIP.

In addition to classifying the biopsies into transcriptomic subclasses, Akbani et al., 2015 had pathologists assess biopsies for lymphocyte infiltration. Biopsies were scored from 0 to 3 on lymphocyte distribution, with 0 indicating no lymphocytes present in the tissue and 3 indicating that lymphocytes were present in over 50% of the tissue. They were also scored from 0 to 3 on lymphocyte density, with 0 indicating an absence of lymphocytes and 3 indicating a “severe” presence. These measures were added to create a Lymphocyte Score, a measure that ranged from 0 to 6 meant to summarize the general presence and degree of lymphocyte infiltration in that biopsy. Of the 235 patients in our sample that were assessed by the pathologists, 29% had a score of zero, 23% had a score of two, 10% had a score of three, 13% had a score of four, 15% had a score of five, and 9% had a score of six. Note that by definition of the component scores, a score of one is not possible. We performed a linear regression of Lymphocyte Score on mean biopsy level logit-CTIP, treating Lymphocyte Score as continuous. We found that a one unit increase in mean biopsy level logit-CTIP was highly significantly associated with a 0.45 unit decrease in mean Lymphocyte Score (*p* ≪ 0.0001). This result is also consistent with both previous results, and provide further evidence for the apparent negative association between mean logit-CTIP and the presence of specific immune cell types generally associated with positive prognosis.

##### 3.2.4.2 Survival analysis

In order to assess association between logit-CTIP and overall survival, we fit a Cox Proportional Hazards model using clinical data as well as CTIP values. Clinical patient data were retrieved from the TCGA website and matched to biopsy images via TCGA identifier. In addition to adjusting for mean logit-CTIP, we adjusted for cancer stage, age in years, and sex. Age in years was standardized by subtracting the mean value and dividing by the standard deviation.

Using this model, we found that after adjusting for other factors an increase in normalized logit-CTIP was significantly associated with increased hazard of death (*p* = 0.02). See Figure 7 for hazard ratio estimates and associated confidence intervals. In addition to this, we found that normalized age was significantly positively associated with an increased hazard of death (*p* < 0.05), as was having stage 3 disease (relative to stage 1, *p* < 0.05). Having stage 4 disease was positively associated with increased hazard of death, though not significantly. This is most likely due to the lack of patients with stage 4 disease in the data set.

In order to assess the performance of CTIP relative to a more traditional measure of spatial association the same model was fitted with average logit-CTIP exchanged for the average value of the estimated G-cross function (discussed previously in Section 3.1) as well as the Mark Connection Function (MCON). The Mark Connection Function is another natural point of comparison because it is a measure that is commonly used to investigate spatial associations between points with discrete marks, it is constrained to lie in [0,1] like CTIP, and it has been used elsewhere as a comparison point for similar survival modeling using spatial information (Li et al., 2019b). To produce a single value, the MCON function was evaluated at *r* = 30, which is the same as the radius of interaction used in our computation of CTIP as well as the G-cross function. Both G-cross and MCON values were logit-transformed. Because the values remained skewed even after logit-transform, they were subsequently centered and scaled by subtracting their respective mean and dividing by their respective standard deviation. While increased normalized logit-MCON was also associated with an increased hazard of death, the association was not significant (*p* = 0.057). The G-cross function was associated with a decreased hazard of death, though the association was also not significant (*p* = 0.32). Figure 7 summarizes the results across all three models.

This result, along with the other results presented in this paper, provide evidence of an immune phenotype in SKCM that corresponds to poor prognosis from a number of different perspectives. With respect to overall survival, we have demonstrated that adjusting for other relevant clinical factors, an increase in CTIP is associated with an increased hazard of death. For gene expression data, average CTIP is associated with decreased expression of genes related to immune cells that are generally associated with good overall prognosis. And finally, we found that average CTIP is significantly lower in biopsies that have been classified as immune enriched through gene expression clustering, as well as biopsies that have been assessed by pathologists to have higher densities and spatial distributions of tumor infiltrating lymphocytes. These results fundamentally support the notion that CTIP is capturing an anti-immunogenic phenotype that is associated with poor prognosis, possibly due to an overall negative association with the presence of immune cells that are associated with improved prognosis such as CD8^+^ T Cells and Natural Killer cells.

## 4 Discussion

The SPARTIN method provides a general framework for applying traditional spatial statistical techniques to whole biopsy imaging data. More specifically, it provides a modular algorithmic mechanism for partitioning the biopsies into more readily analyzable sub-regions that can be assessed for local spatial patterns using marked point process models. We have demonstrated the utility of such a method using a novel characterization of spatial cellular interaction, CTIP, that allows rigorous quantification of uncertainty and is highly intepretable. We demonstrate through simulation that CTIP can reliably distinguish between different patterns of spatial interaction across a range of different dependencies. We also demonstrate the utility of SPARTIN through a comprehensive analyses of an SKCM dataset, wherein we quantify and evaluate the spatial extent of tumor-immune infiltration and its association with genomic, phenotypic and clinical outcomes. These results are particularly suggestive in the context of SKCM. Melanoma has a uniquely rich literature regarding the potential applications of immunotherapy. While there are many factors that have contributed to this, not the least of which is the relative accessibility of skin cancers compared to other types of cancer, the net result is a far more mature understanding of immunotherapy in the context of melanoma than other types of cancer (Oble et al., 2009; Nguyen et al., 2010; Pitcovski et al., 2017). Thus, possible implications of the spatial immune organization in melanoma for immunotherapy may be more readily interpreted within the context of Melanoma than other cancer types, particularly as the ability to distinguish between different types of immune cells improves.

The SPARTIN pipeline represents a valuable contribution in and of itself to digital pathology through its ability to model and quantify immune infiltration across entire biopsies in a way that captures meaningful variation along with uncertainty quantifications across patients. However, it also creates numerous opportunities for future work in this area. As algorithms for cell-level image classification improve, the opportunities for more and more detailed quantitative analysis of histopathological imaging data will become both more numerous and more fruitful. Specifically, as the ability to reliably distinguish between different types of immune cells will allow for more granular investigations into the prognoses associated with interaction between immune cells of different kinds and the tumor. Moreover, as spatial biology techniques (e.g., spatial multiplex imaging and spatial transcriptomics) evolve and data becomes abundant, so too will opportunities for synthesizing data on the relative spatial locations of different cell types along with cell or spot specific gene/protein expression data through rigorous, principled, and complex modeling. It would be straightforward (particularly in a Bayesian framework) to model a function of one or all of the parameters of interest as a linear combination of the local genes/proteins that are known to be relevant to immune response. Alternatively, future methods in this area could be developed at the intersection of spatial data and high-dimensional omic features.

The SPARTIN pipeline could also be generalized for further investigation not directly related to assessing immune infiltration. Partitioning each biopsy into non-overlapping sub-regions invites the application of other tools from the spatial statistical catalogue to other relevant spatial features of the tumor microenvironment. In fact, SPARTIN can be utilized even when investigating features of the tumor that are not spatial in nature. So long as a feature (such as average tumor cell size) can be quantified at the cellular level and summarized locally at the level of sub-regions, SPARTIN provides a framework for mapping the variation of that feature across the entire biopsy.

One limitation of this analysis is that immune cells were modeled as a single agglomerated class rather than as separate cell types belonging to the same family. While our results paint a consistent picture from several different angles, the exact mechanism of these various associations could be further refined by utilizing a more precise categorization of the various types of immune cells. It is worth emphasizing, however, that nothing about the structure of our pipeline depends on the presence of only one type of immune cell in addition to the cancer cells. The SPARTIN method can be generalized to multiple types cells through multicategory marked point process models, though this would raise additional computational and practical challenges. Another potential limitation of our analysis that suggests an avenue for future methodological development is the presence of repeated measurements within biopsies. Note that the result of fitting models on the partitioned tumor microenvironment is a set of repeated observations with some underlying spatial covariance structure related to the spatial relationships between the different subspaces. For now, we have ignored this spatial covariance, and instead chosen to summarize the variation at the biopsy level. While this does take into account local spatial dependencies (i.e., within tiles), it is possible that incorporating additional global spatial structure across the biopsy (i.e., between tiles) could lead to additional gains in efficiency, and possibly insight into the nature of the spatial variation in immune activation in the tumor microenvironment. Thus, future work will likely include the development of methods to efficiently analyze this complex structured areal data. Finally, it should be noted that the hierarchical Strauss model makes two important assumptions about the nature of the data, those of stationarity and isotropy. This essentially amounts to the assumption that on the level of the tiles for each biopsy on which the models are fit, the point process would be unaffected by horizontal translation (stationarity) or rotation (isotropy). Because tumor biopsies do not have a natural orientation in $R^{2}$ , it is plausible to assume isotropy. Stationarity is somewhat more challenging to justify *a priori*. However, because the model fitting takes place on relatively small sub-sections of the biopsy, i.e., the “tile” specific level, it is quite plausible that this assumption is at least locally satisfied well enough. Since each tile is modeled using separate spatial parameters, this accounts for non-stationarity at a biopsy level and the ultimate measure of interest, CTIP, still yields a reliable estimate of the degree of interaction between cell types. Still, future work could involve employing methods that relax or eliminate these assumptions during the modeling of interaction.

Software is available at https://github.com/bayesrx/SPARTIN. The website for the project, which features an application that can be used as a visualization companion to the software can be found at https://nateosher.github.io/SPARTIN. Using the SPARTIN R package, data can be exported and visualized on the web application in order for users to interactively navigate the patterns of interaction at both the biopsy and individual tile level.

## Data Availability

The datasets presented in this study can be found in online repositories. The names of the repository/repositories and accession number(s) can be found in the article/Supplementary Material.
