# Supplementary material for: SPARTIN: a Bayesian method for the quantification and characterization of cell type interactions in spatial pathology data
Source: Front Genet. 2023 May 18;14:1175603. doi: 10.3389/fgene.2023.1175603 (PMC10232864; doi:10.3389/fgene.2023.1175603)
Supplement: Supplementary file 1 [file Presentation1.pdf]

Supplementary Material

1 DATA SUMMARY

Our data set contained 335 SKCM biopsies. The cell counts varied by biopsy, both in terms of the overall numbers and the specific numbers of tumor cells and immune cells. See Figures S1, S2, and S3 below.

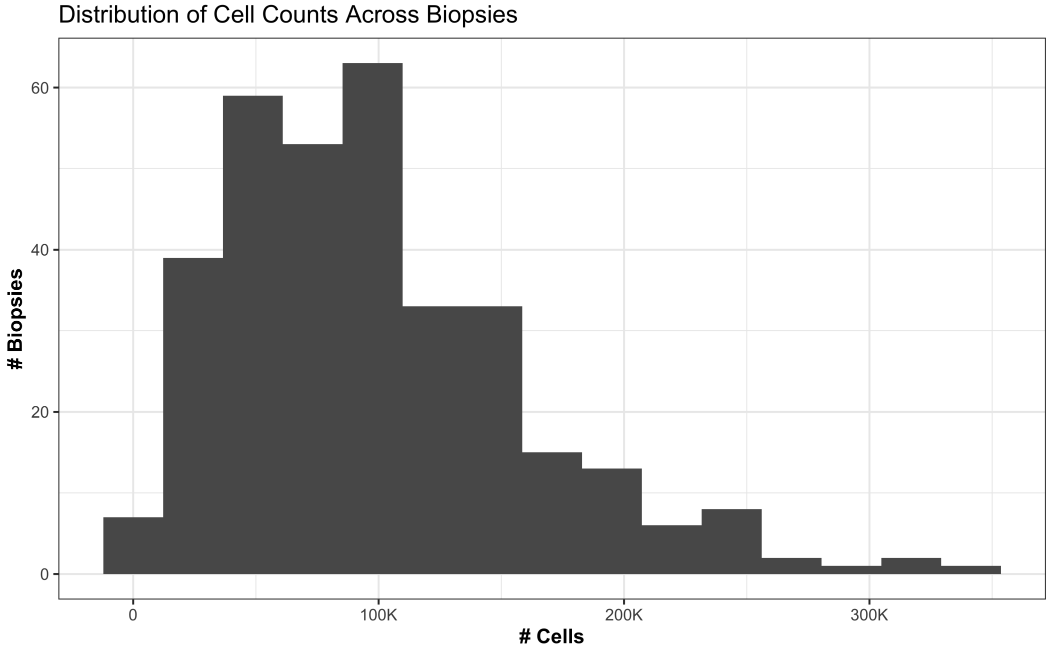

Figure S1: *Distribution of cell counts.* Distribution of total number of cells across all biopsies.

2 MODEL FITTING

Given a quadrature $\boldsymbol{u}$ on A with corresponding weights $\boldsymbol{w}$, the pseduolikelihood function (equation (3) in the main paper) can be approximated by

$$PL(\theta|\boldsymbol{x}) \approx \prod_{x_i \in \boldsymbol{x}} \lambda(x_i|\theta, \boldsymbol{x}) \exp(-\sum_{u_j \in \boldsymbol{u}} \lambda(u_j|\theta, \boldsymbol{x}) w_j) \tag{S1}$$

The density of the selected quadrature $\boldsymbol{u}$ on A is chosen to balance the accuracy of the approximation against the computational load that larger quadratures impose.

Using this approximation of the pseudolikelihood function in place of the more standard likelihood function, Bayesian analysis can proceed in the style of King et al., 2012 by simply assigning priors to the parameters of interest and using techniques to sample from non-closed form posterior likelihoods. We assigned non-informative normal priors with mean 0 and variance 10^6 to γ_{12}, β_1, and β_2. In all analysis presented in the main paper the quadrature and weights used to estimate the integral in the pseudolikelihood

function was generated by the spatstat package (Baddeley and Turner, 2005). Samples from the posterior were taken using JAGS via the R2jags package (Su et al., 2015).

In order to use JAGS to fit the model, the so-called "Poisson zero trick" as described in Kruschke, 2014. This allows for the fitting of a model with an arbitrary likelihood (or pseudolikelihood) function. The zero trick works by explicitly specifying the log-likelihood function of the model to be fit for each observation, and treating the resulting log-likelihood as the rate parameter of a Poisson distribution. Note that because the log-likelihood function can be negative, a constant value may have to be added in order to ensure the resulting value is strictly positive, since the rate parameter of a Poisson distribution must be strictly positive. But if the same value is used for all observations in a single model fitting across iterations, this is equivalent to multiplying the likelihood by a constant, which does not affect the inference. A vector of zeros is passed to JAGS, and each zero is said to be observed from a Poisson distribution with rate parameter specified by the modified log-likelihood function evaluated for that observation. By definition of the probability mass function of the Poisson distribution, the resulting likelihood is the target likelihood for each observation. An analogous trick using the Bernoulli distribution and a vector of ones can be used as well- see Kruschke, 2014 for details. Briefly, denoting the number of observed points n_o, the number of quadrature points n_q, and $n_o + n_q = N$, the underlying MCMC algorithm is as follows:

Algorithm 1 Bayesian Strauss Model Fitting

for biopsy $i = 1, \ldots, n\_iter$ **do**
  for $\theta = \beta_1, \beta_2, \gamma_{12}$ **do**
    Propose θ^*
    Evaluate $PL(\theta^*|\cdot)$
    Set $\theta^{(i)} = \theta^*$ with probability $min(\frac{PL(\theta^*)}{PL(\theta)}, 1)$;
otherwise, set $\theta^{(i)} = \theta$
  end for
end for
return $\beta_1^{(1)}, \ldots, \beta_1^{(n\_iter)}, \beta_2^{(1)}, \ldots, \beta_2^{(n\_iter)}, \gamma_{12}^{(1)}, \ldots, \gamma_{12}^{(n\_iter)}$

Results were checked against both frequentist model fittings from the spatstat "ppm"/"hierstrauss" functions as well as STAN model fittings (Carpenter et al., 2017) and yielded virtually indistinguishable results in both cases. Despite STAN sampling more quickly than JAGS, JAGS still outperformed STAN due to its somewhat shorter time in setting up the model for sampling. Using JAGS, for a tile with 75 tumor cells, and 327 immune cells, 2,000 burn-in samples, 2,000 post-burn-in samples, and 5 null simulations, CTIP takes roughly 2 minutes to compute on the corresponding author's machine (2020 13-inch Macbook Pro, M1 Processor, 8 GB of RAM).

3 BIOPSY PARTITION

In order to partition a given biopsy into non-overlapping sub-regions, we began with the smallest bounding rectangular window that contained all cells. We then applied an intensity thresholding algorithm in order to find the smallest possible window that still contained virtually all cells. The purpose of intensity thresholding is to remove excess white space from around the biopsy. If the biopsy is not intensity thresholded, the resulting white space biases the model estimates, since we are essentially treating this space as an area where we might conceivably observe cells, when in reality there are none. Intuitively,

this inflates the apparent spatial association, since the estimated value of the interaction parameter must increase to compensate for the lowered estimate of first order intensity that results from increasing the size of the window without any increase in the number of points. This was accomplished using the "density.ppp" function from the "spatstat" package (Baddeley and Turner, 2005). The bounding rectangular window was broken up into small ($15\mu m \times 15\mu m$) pixels, the intensity of which was estimated as a function of the number of points per square unit of area of the pixel. These values were also smoothed between pixels according to a pre-selected bandwidth of $25\mu m$ chosen through experimentation. The final window was constructed by combining all pixels that were above a certain intensity into a window. The "pixel" size, smoothing bandwidth, and intensity threshold collectively determined the final window, and the same settings ($15\mu m^2$ pixel size and $25\mu m$ smoothing bandwidth) were applied across all biopsies.

4 CELL CLASSIFICATION MODEL DATA

During the annotation and labelling stage, the classification paradigm focuses on the "diversity" of cells being annotated; that is, we try to capture as many different "presentations" of each cell type in the image dataset. Additionally, the classification is done based on the morphological and intensity paradigm of each annotation object, which in this case is one given cell. Because each labelling instance is treated independently, the imbalance in the occurrence of cell types across different biopsy images should not significantly impact the classification performance.

**Class**	Tumor (1)	Immune (2)	Other Cells (3)	Macrophage (4)	**Total Cells**
**Number of Labels**	288	349	252	361	1,250

Table S1. *Training data cell composition.* Cell type composition of training dataset.

5 ADDITIONAL SIMULATION RESULTS

we have run a small scale simulation study using a modified version of the method in the paper. Briefly, the simulation compares two settings. In the first setting, there is positive interaction *within* the two cell types, but no interaction *between* them. We accomplished this by simulating cells of each type to be within $r = 30$ of another existing cell of that type with probability 0.5, or to be drawn from a poisson process with probability 0.5. Thus, each type of cell has a tendency to be near cells of its own type, but no systematic spatial relationship between cells of the other type. In the second setting, there is positive interaction both within *and* between the two cell types. We accomplished this by modifying the first simulation to have an additional step: if a cell is not simulated to be interacting with a cell of its own type, it is then simulated to be interacting with a cell of the other type with probability 0.3; otherwise, it is drawn from a Poisson process. All simulated data sets consist of fifty simulated tumor cells, and fifty simulated immune cells. Fifty data sets were simulated for both settings, and a value of CTIP (our method) and G-cross (comparative method) evaluated at $r = 30$ was estimated for all simulations.

S5 and S6 show the resulting distributions of CTIP and G-cross in the two settings, as well as the corresponding ROC curves. The values of CTIP in setting 2 are clearly on average noticeably higher than those of setting 1, whereas there is substantially more overlap in the distribution of G-cross across the two settings. It therefore seems CTIP can identify degrees of positive interaction between classes even when positive interaction within classes is present. It should also be noted that unlike the previous simulation

method, this method also explicitly violates the assumption of hierarchy between the two types of points: the simulated tumor cells can influence the locations of the simulated immune cells, but the simulated immune cells can also influence the locations of the simulated tumor cells. Moreover, note that in the second setting, *within* class interaction takes "precedent" over between class interaction, i.e. simulated cells always have the possibility of interacting with a cell of their own type, and only if they are selected not to interact with a cell of the same type do they have the possibility of interacting with a cell of the other type. The ROC curve was computed by treating the true classification of every simulation in setting 1 as non-positive interaction and every simulation in setting 2 as positive interaction.

6 TESSELLATION SENSITIVITY ANALYSIS

6.1 Tile Size

We examined the impact of shifting the size of the tiles on the biopsy with the median number of tumor cells. The original partition was performed to achieve roughly 75 tumor cells per tile, with a maximum of 100 and a minimum of 50. In the sensitivity analysis we experimented with partitioning the biopsy into smaller tiles (roughly 50 tumor cells per tile, maximum 75, minimum 25), and larger tiles (roughly 100 tumor cells per tile, maximum 125, minimum 75). We then computed CTIP for all tiles in the resulting tessellations. Figures S7 and S8 summarize the results. Figure S7 shows the actual results of the different tessellations, along with the estimated value of CTIP by tile. Figure S8 shows the distribution of tumor cell counts by tile for each of the tessellations and the distribution of CTIP across the entire biopsy for each tessellation, respectively. As can be seen, this does not significantly change the distribution of CTIP on the biopsy. Perhaps more importantly, the means across these three settings are 0.693 for the small tile tessellation, 0.701 for the actual tessellation, and 0.716 for the large tile tessellation. This demonstrates the robustness of our method to small scale perturbations in the tile size and partitions.

6.2 Clustering Algorithm

To examine sensitivity to the clustering algorithm used, we have repeated the analysis of the same biopsy using a different clustering algorithm, which we refer to as ”greedy clustering.” To create clusters using this algorithm, we used the following process:

1. Of the non-clustered cells, select the cell with the smallest x-value
2. Of the other non-clustered cells, select the 74 nearest cells; group into cluster
3. Remove the newly clustered cells from consideration
4. Repeat $(1) - (3)$ until no cells remain, or fewer than the minimum possible cluster size (50) remain

This algorithm differs considerably from the K-means algorithm used in the actual analysis, as do the resulting clusters. Figure S9 shows the results of this tessellation along with the estimated values of CTIP for each tile, the actual tessellation used in the analysis, and a comparison between the distributions across the two tessellations. The mean CTIP for the actual tessellation is 0.701, and the mean for the greedy tessellation is 0.710, which does not suggest a problematic degree of sensitivity to the clustering algorithm used.

REFERENCES

Ruth King, Janine B Illian, Stuart E King, Glenna F Nightingale, and Ditte K Hendrichsen. A bayesian approach to fitting gibbs processes with temporal random effects. *Journal of agricultural, biological, and environmental statistics*, 17(4):601–622, 2012.

Adrian Baddeley and Rolf Turner. Spatstat: an r package for analyzing spatial point patterns. *Journal of statistical software*, 12:1–42, 2005.

Yu-Sung Su, Masanao Yajima, Maintainer Yu-Sung Su, and JAGS SystemRequirements. Package 'r2jags'. *R package version 0.03-08, URL http://CRAN. R-project. org/package= R2jags*, 2015.

John Kruschke. Doing bayesian data analysis: A tutorial with r, jags, and stan. 2014.

Bob Carpenter, Andrew Gelman, Matthew D Hoffman, Daniel Lee, Ben Goodrich, Michael Betancourt, Marcus Brubaker, Jiqiang Guo, Peter Li, and Allen Riddell. Stan: A probabilistic programming language. *Journal of statistical software*, 76(1), 2017.

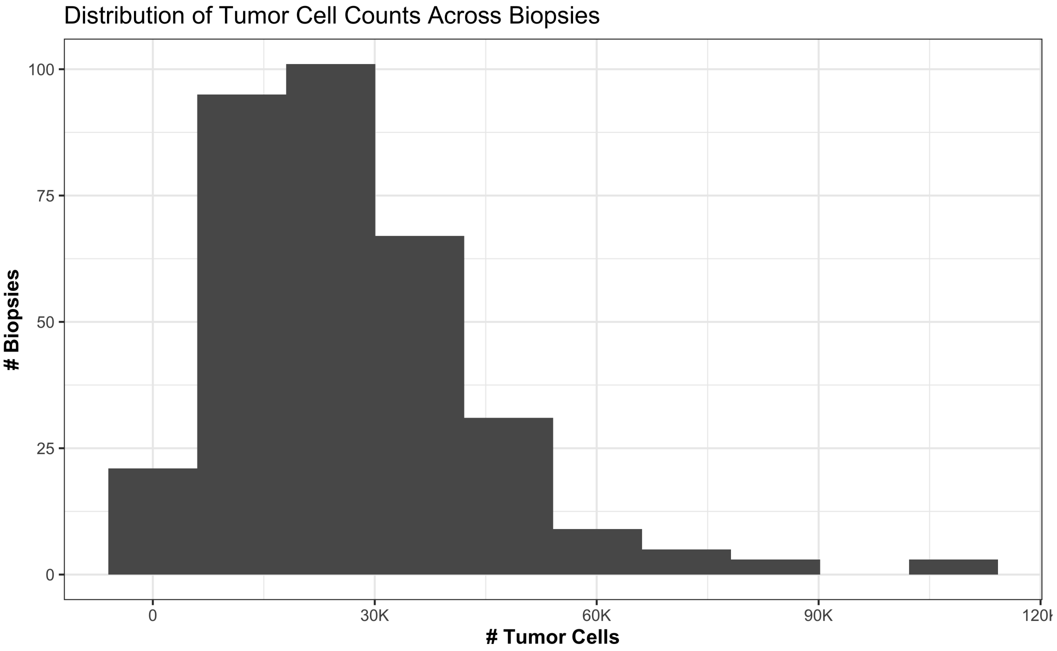

Figure S2: *Distribution of tumor cell counts.* Distribution of total number of tumor cells across all biopsies.

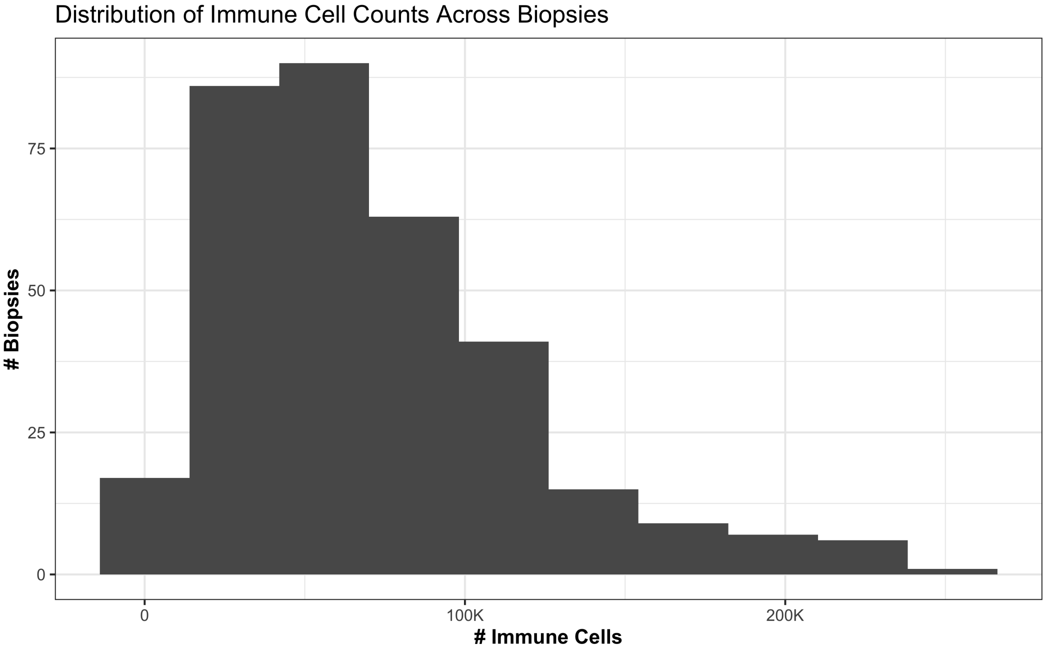

Figure S3: *Distribution of immune cell counts.* Distribution of total number of immune cells across all biopsies.

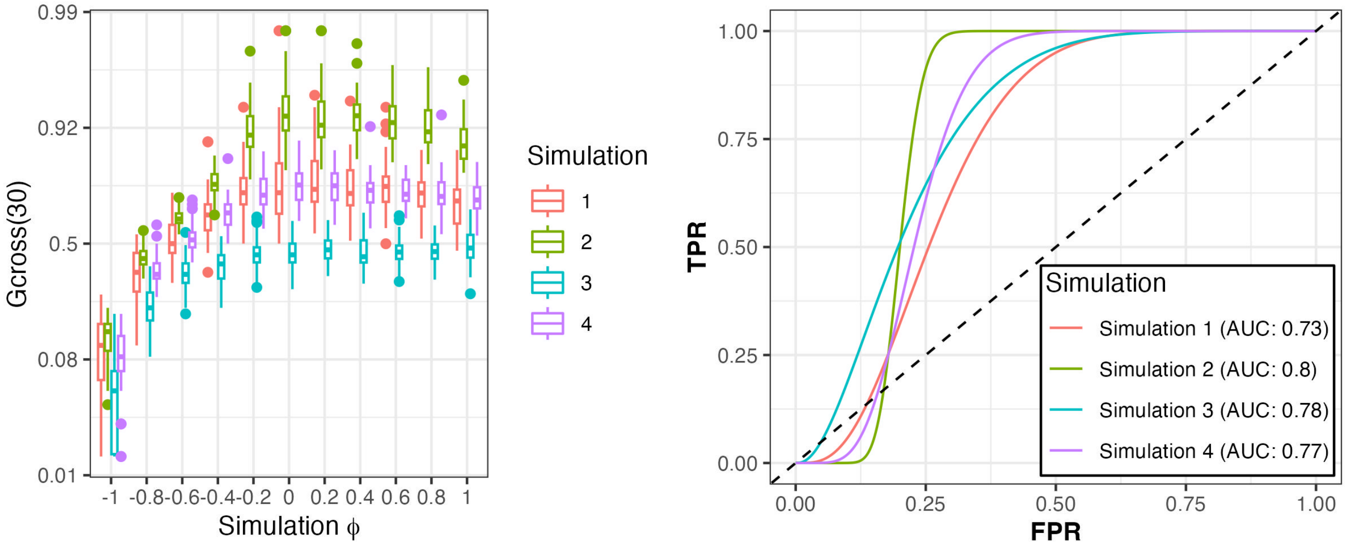

Figure S4: *Classification performance using G-cross function evaluated at 30 micron distance.* Left: distributions of G-cross function evaluated at 30 microns across different simulation settings and values of ϕ. Right: ROC curves and associated AUC values across different simulation settings.

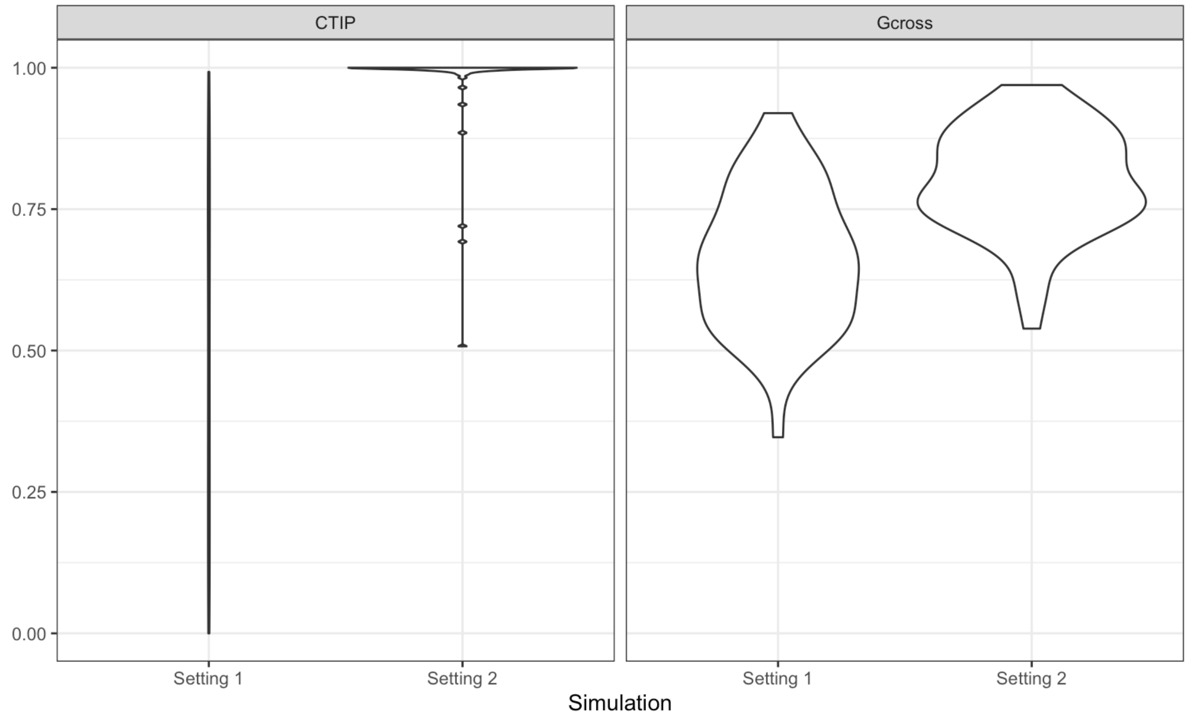

Figure S5: Distribution of CTIP and G-cross across additional simulation settings. Setting 1 had interaction between cells of the same type, but no interaction between the two types of cells; setting 2 had interaction both within and between cells of both types.

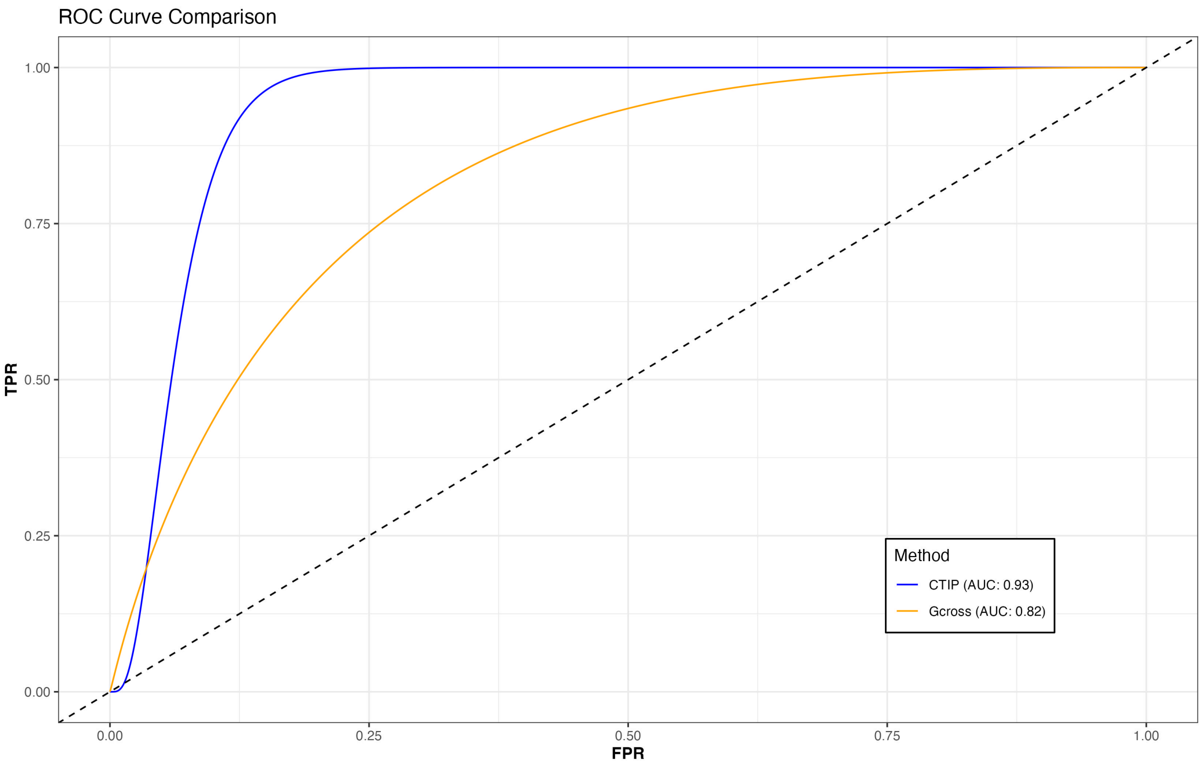

Figure S6: ROC curves for CTIP and G-cross in additional simulations. Simulations in setting 1 were classified as having non-positive interaction, while simulations in setting 2 were classified as having positive interaction.

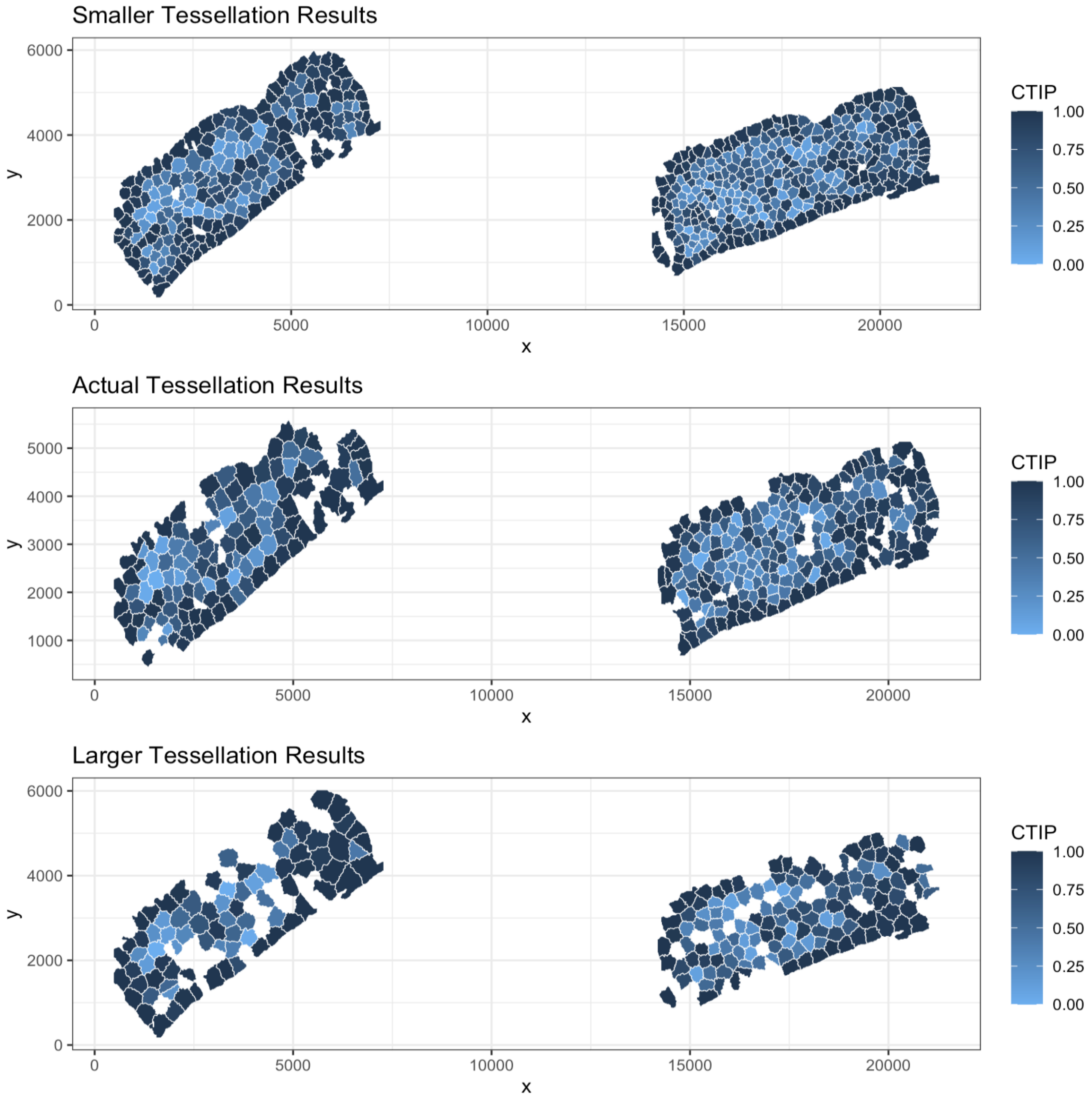

Figure S7: Results of tessellations with different tile sizes. Top: small tiles, 25-75 tumor cells per tile. Middle: actual tessellation, 50-100 tumor cells per tile. Bottom: large tiles, 75-125 tumor cells per tile.

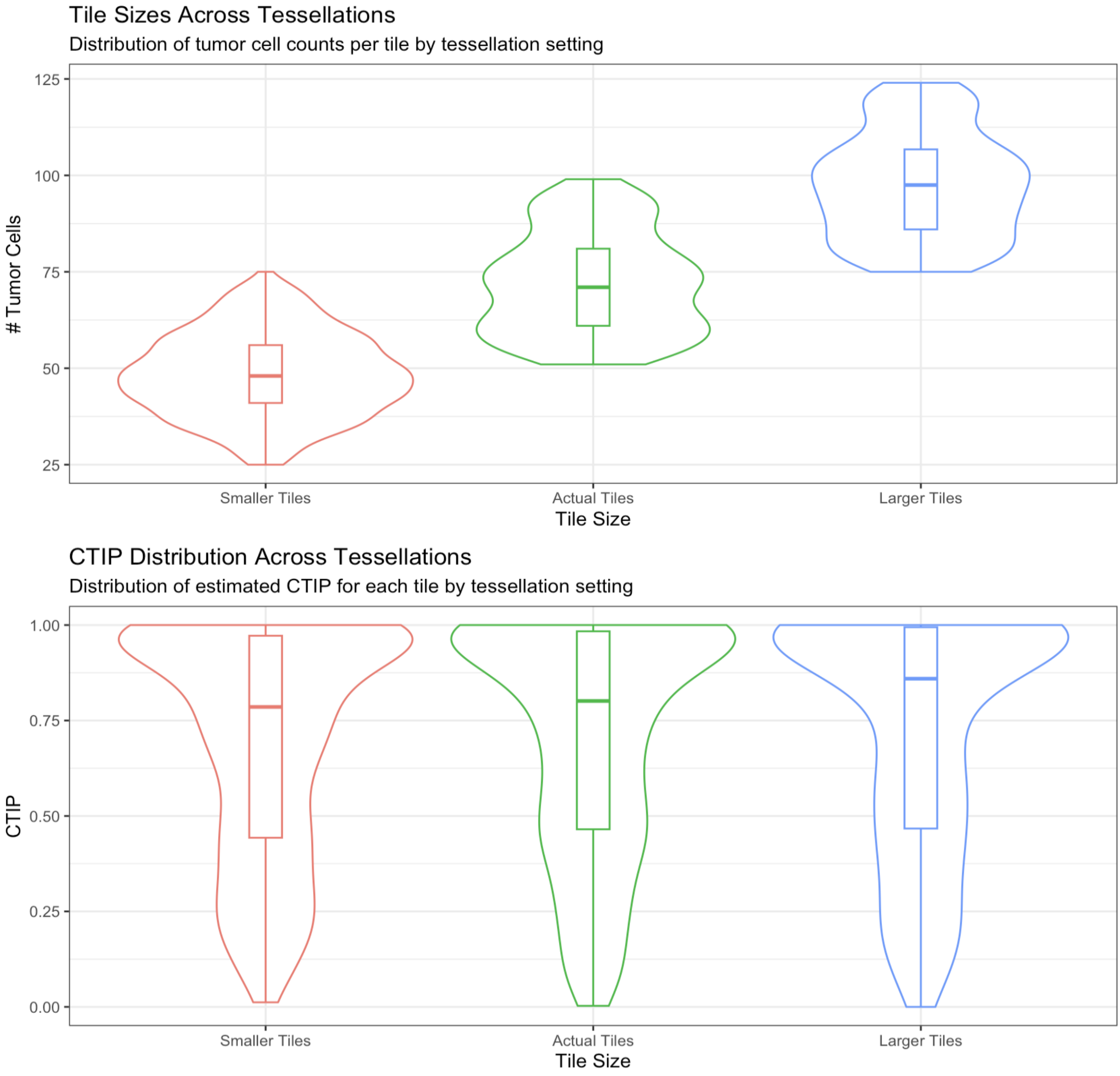

Figure S8: Top: distribution of tumor cell counts per tile across three tessellation settings. Bottom: distribution of CTIP across different tessellation settings.

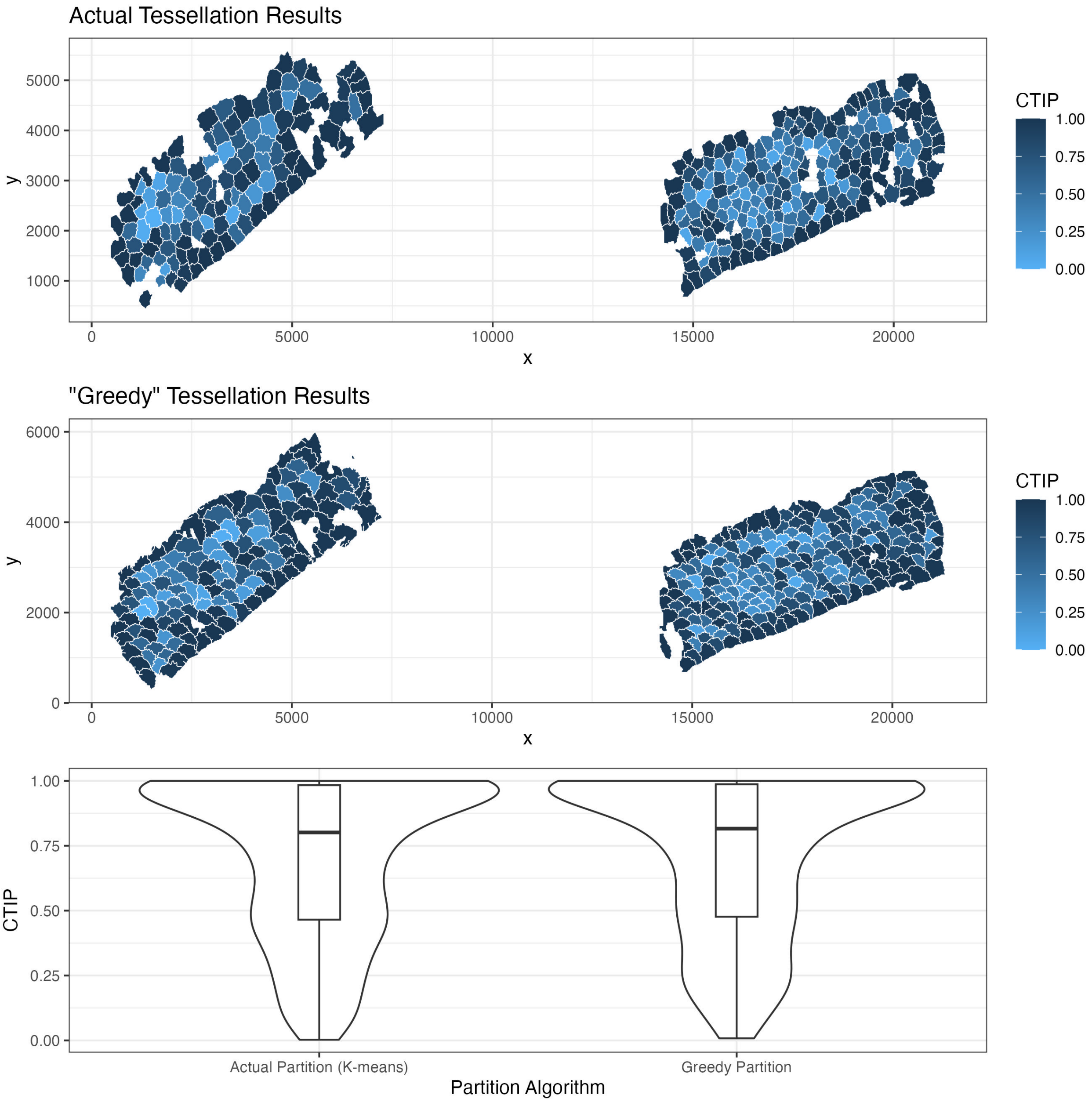

Figure S9: Results of tessellation using "greedy" algorithm. Top: actual tessellation and values of CTIP used in analysis. Middle: Tessellation and values of CTIP using greedy partition. Bottom: Comparison of distributions of CTIP across the two tessellation methods.
